# Sensory Properties and Main Differential Metabolites Influencing the Taste Quality of Dry-Cured Beef during Processing

**DOI:** 10.3390/foods11040531

**Published:** 2022-02-12

**Authors:** Huihui Fu, Li Pan, Jingyun Wang, Jixing Zhao, Xin Guo, Jingya Chen, Shiling Lu, Juan Dong, Qingling Wang

**Affiliations:** 1 Laboratory of Meat Processing and Quality Control, College of Food Science and Technology, Shihezi Univesity, Shihezi 832000, China; fuhui2299@163.com (H.F.); panl13116@163.com (L.P.); wang20220125@163.com (J.W.); zhaojixing_2016@126.com (J.Z.); guoxin0317edu@163.com (X.G.); c13070397276@163.com (J.C.); lushiling_76@163.com (S.L.); djshzu@126.com (J.D.); 2 College of Cooking and Catering Management, Xinjiang Vocational University, Urumqi 830013, China

**Keywords:** dry-cured beef, metabolomics, sensory assessment, taste compounds, correlation analysis

## Abstract

This study adopted widely targeted high-performance liquid chromatography-tandem mass spectrometry (HPLC-MS/MS) metabolomics and multivariate data analysis methods to evaluate the correlation between changes in metabolites and their taste formation in dry-cured beef during processing. The physicochemical profile changed significantly in the maturity period (RG), especially due to the continuous hydrolysis and oxidation of proteins. The sensory characteristic of dry-cured beef was highest in saltiness, umami, overall taste, and after-taste in RG. Overall, 400 metabolites were mainly identified, including amino acids, peptides, organic acids, and their derivatives, nucleotides, and their metabolites, as well as carbohydrates. Cysteine and succinic acid were significantly up-regulated during the process of dry-curing beef compared to the control group (CG). Moreover, glutamine and glutathione were significantly down-regulated in the fermentation period (FG) and in RG. Kyoto Encyclopedia of Genes and Genomes (KEGG) enrichment analysis revealed that glyoxylate and dicarboxylate metabolism, glutathione metabolism, alanine, aspartate, and glutamate metabolism, arginine biosynthesis, taurine, and hypotaurine metabolism were the main metabolic pathways influencing the taste of dry-cured beef during processing. Results of correlation analysis revealed that umami is positively correlated with salty, L-cysteine, L-arginine, inosine, creatinine, and succinic acid. Our study results provide a better understanding of the changes in taste substances and will contribute to quality evaluation of dry-cured beef.

## 1. Introduction

Dry-cured beef is a traditionally dry-cured meat product in Xinjiang. It is produced from fresh beef by dry-curing, fermentation, and ripening. It is highly preferred by consumers because of its unique flavor, delicious taste, good storage, and other features. Xinjiang is very conducive to the processing of dry-cured beef, since it has excellent natural pastures at high altitudes. Being produced in this special climatic condition brings dry-cured beef tenderness, good palatability, and green and pollution-free characteristics [1]. Taste is one of the critical characteristics to influence edible quality meat products among the sensory properties. Taste is mainly derived from the primary and secondary metabolites in the processing of dry-cured meat. This is because non-volatile compounds formed during processing may have a positive effect on the taste of the final product and eventually determine product choice by consumers [2].

Previous studies have reported that several flavor precursors with low molecular weight that contribute to the formation of quality (texture and flavor) are produced when processing dry-cured meat products. They are produced as a result of protein hydrolysis, oxidation, and degradation processes [3,4]. In addition, non-volatile flavor precursors including amino acids, creatine, carbohydrates, and nucleotides can also affect the taste, flavor, tenderness, and juiciness of meat products [5,6,7,8]. Several studies [9,10,11] have shown that water-soluble compounds of low molecular weight not only act as flavor precursors, but also have taste characteristics. Koutsidis et al. [12] reported that free amino acids, carbohydrates, organic acid, and peptides in stored beef muscle contribute to umami, sweet, sour, and bitter taste. Meanwhile, continuous accumulation of water-soluble flavor precursors increases flavor intensity. According to experiments conducted by Lou et al. [13] on vinasse-cured duck, it was found that amino acids, carbohydrates, alkaloids, organic acids, and nucleotides as well as their derivatives are the main taste substances in meat products. Moreover, Zhou et al. [14] reported that free amino acids hydrolyzed from proteins by endogenous enzymes and exogenous enzymes are the primary non-volatile components in dry-cured goose meat. However, the reports on the taste quality of dry-cured beef are still limited, and there is still no study that has evaluated the production of non-volatile components.

As a comprehensive metabolite profiling technology, metabolomics is a new tool used for quality assessment of meat products. It refers to the systematic analysis of endogenous metabolites and their dynamic changes in a specific tissue or organism after environmental stimulation in a qualitative and quantitative manner [15]. The ‘metabolome’ represents a collection of all metabolites in a system. Among the metabolites are reactants, intermediates, and end-products of the metabolism [16]. Several different analytical techniques are used to measure metabolites, while nuclear magnetic resonance (NMR) [2], gas chromatography-mass spectrometry (GC–MS) [17], and liquid chromatography-mass spectrometry (LC–MS) [18] are the most commonly used. Nuclear magnetic resonance-based metabonomics analysis of the metabolic mechanisms of boneless dry-cured hams have shown that amino acids, organic acids, and nucleotide derivatives are the major contributors to taste [19]. A study carried out by Zhang et al. [20] distinguished different types of dry-cured ham using the map of major metabolites. Elsewhere, Sugimoto et al. [11] used capillary electrophoresis-mass spectrometry. According to their study, the taste of dry-cured ham samples matured at 540 days was the best with the highest score during sensory evaluation. Another related study used multivariate data analysis techniques to visualize correlation patterns among metabolites of chickens [5]. However, few studies have focused on the metabolites’ profiles of dry-cured beef during processing [1,21,22].

It is essential to comprehensively analyze the variation of metabolites during processing to acquire a better understanding of the taste formation process and further assess the quality of dry-cured beef. Therefore, the present study was conducted to determine the metabolite profiles of dry-cured beef samples with raw material, fermentation period, and maturity period using HPLC-MS/MS coupled with multivariate analysis. The results of this study will provide a theoretical research foundation for the technology promotion and improvement of dry-cured beef processing, thereby contributing to its enhanced industrialization process.

## 2. Materials and Methods

### 2.1. Materials

A fresh beef loin was purchased from a local farmers’ market for 3-year-old Xinjiang brown cattle carcasses fed by Shihezi Tiankang Animal Husbandry Biotechnology Co., Ltd. (Shihezi, China). Cattles were fed casually with fodder based on barley, bran, corn, sorghum, potatoes, and feed chalk, and raised together.

Eighteen beef loins were used in this study, where each loin was taken from each carcass, and cut equally into three pieces. The average weight of each piece was about 2.00 ± 0.05 kg. A total of 54 pieces were used for experiments. Dry-cured beef was produced in the Animal Product Processing Laboratory of Shihezi University (Xinjiang, China), during a 5-month processing period.

### 2.2. Processing of Dry-Cured Beef

The beef samples were salted for one month at a constant temperature and humidity room (8 °C and 65% relative humidity RH). During the curing period, the beef was pickled five times with different salt concentrations of 2.0, 2.0, 1.0, 0.5, and 0.5%, respectively, on the 1st day, the 4th day, the 8th day, the 13th day, and the 19th day, where the raw meat samples were manually rubbed with salt (2% of the weight of the meat) until the salt dissolved. Then they were kept at a constant temperature and humidity cabinet to salt. After salting, the samples were washed and then kept in a drying room at 10 °C and 55% RH for 30 days. Samples were then kept in a fermenting room at a temperature of 20 °C and 70% RH for 30 days. They were then exposed to a temperature of 23 °C and 75% RH for 30 days. Finally, the beef samples were placed in a ripening room at 25 °C and 70% RH for 30 days.

Three processing points were selected at different periods: CG—control group, FG—fermented group for 120 days, as well as RG—ripened group for 150 days. Each group was replicated six times. Samples were vacuum-packed and frozen at −80 °C for experimental analysis.

### 2.3. Physicochemical Properties and Protein Hydrolysis and Oxidation

The moisture and sodium chloride contents of each dry cured beef sample collected in the different processing stages were determined using the methods described in Association of Official Analytical Chemists (AOAC) 984.25 and AOAC 935.47 [23], respectively. The pH values were determined with a pH meter (PHS-3C, Shanghai INESA Scientific Instrument Co., Ltd., Shanghai, China). The total protein content of different processing samples was measured by the Kjeldah method and the non-protein nitrogen content was measured according to Careri et al. [24], and the proteolytic index (PI) was the ratio of non-protein nitrogen to total nitrogen. Myofibrin (MP) and sarcoplasmic protein (SP) were extracted from samples of different periods according to the methods of Ran et al. [25]. MP and SP were diluted to 1 mg/mL and left to react with 2, 4-dinitrophenylhydrazine, and the absorbance was measured at 370 nm to obtain the content of the carbonyl group [26], and after the reaction with 2-nitrobenzoic acid, the absorbance was measured at 412 nm to calculate the total sulfhydryl group content [27], respectively.

### 2.4. Sensory Assessment

For sensory assessment, this study used the protocol as described by Yang et al. [28], but with slight modifications. The dry-cured beef samples were cut into slices (about 2 × 2 × 1 cm) and placed into the steamer, where they were steamed for 15 min. The slices were then placed in numbered disposable plates and presented to a panel of judges specializing in sensory assessment and sensory analysis. The panel was composed of five males and five females (aged 20–30 years old). Each panel member was then requested to rate the taste characteristics (sweetness, saltiness, umami, bitterness, sourness, after-taste, and overall taste) of dry-cured beef samples on a scale of 0 (tasteless) to 5 (strong taste). Panelists were asked to whirl the samples in their mouths for 10 s and spit out the samples afterward. To avoid fatigue and lingering effects, the panelists were asked to gargle at least twice with ultrapure water and rest for 2 min.

The evaluation was carried out in the process processing room of the Animal Products Processing Control and Safety Center of Shihezi University, Shihezi City, Xinjiang. Sensory evaluation was carried out under controlled light, temperature, and humidity. Sensory descriptors were defined as follows: sourness (taste on the tongue associated with citric acid), bitterness (taste on the tongue associated with caffeine), sweetness (taste on the tongue associated with sucrose), umami (taste on the tongue associated with monosodium glutamate), saltiness (taste on the tongue associated with sodium and chloride ions), aftertaste (taste remaining in the mouth after product was tasted, chewed, and then expelled), and overall taste (the combination of different tastes).

### 2.5. Analysis of Dry-Cured Beef Metabolomics Using UPLC-MS/MS

#### 2.5.1. Sample Preparation

Samples were thawed on ice, and then a 50 mg sample was placed into an Eppendorf (EP) tube, and afterwards, cold steel balls were added to the mixture. The sample was then homogenized at 30 Hz for 3 min using a MixerMill MM 400 (Retsch Technology, Haan, Dusseldorf, Germany). One mL of 70% methanol with internal standard extract ([2H3]-L-Carnitine HCl, 4-Fluoro-L-α-phenylglycine, L-Phenylalanine (2-13C), L-2-chlorophenylalanine, [2H5]-Kynurenic Acid, [2H5]-Hippuric Acid, [2H5]-Phenoxy acetic Acid) was added, and the mixture was vibrated for 5 min, then centrifuged (8064× *g*) at 4 °C for 10 min. After centrifugation, 400 μL of the supernatant were transferred into the EP tube and stored at −20 °C overnight. This was followed by centrifugation (8064× *g*) at 4 °C for 3 min. Finally, 200 μL of the supernatant were transferred into the liner of the injection bottle for on-board analysis. The quality control samples (QC) were prepared by mixing sample extracts, and were used to demonstrate the precision of the assay under the same processing method. During instrumental analysis, a quality control sample was inserted into every 10 test analysis samples to monitor reproducibility of the analysis process.

#### 2.5.2. High-Performance Liquid Chromatography-Mass Spectrometry and Qualitative and Quantitative Analysis of Metabolites

The sample extracts were analyzed using an UPLC-ESI-MS/MS system (UPLC, Shimadzu Nexera X2, MA, USA; MS, Applied Biosystems 6500 QTRAP, Framingham, MA, USA). The Waters ACQUITY UPLC HSS T3 C18 (100 mm × 2.1 mm × 1.8 µm) chromatographic column was used with the following liquid chromatography conditions: column temperature was 40 °C, constant flow rate was at 0.4 mL/min, and the injection volume was 5 µL. Samples were rapidly eluted using 0.1% formic acid in water (solvent A) and 0.1% formic acid in acetonitrile (solvent B). The separation was achieved with the gradients of 95:5 *v*/*v* (solvent A/solvent B) at 0 min, 10:90 *v*/*v* at 10.0 min, 10:90 *v*/*v* at 11.0 min, 95:5 *v*/*v* at 11.1 min, and 95:5 *v*/*v* at 14.0 min.

The effluents were alternatively connected to an ESI (electrospray ionization)-triple quadrupole-linear ion trap MS/MS (ESI-Q TRAP-MS/MS). LIT and triple quadrupole (QQQ) scans were acquired on a triple quadrupole-linear ion trap mass spectrometer (QTRAP), API 6500 QTRAP LC-MS/MS System, equipped with an ESI Turbo Ion-Spray interface operating in positive and negative ion mode and controlled using the Analyst 1.6.3 software (Sciex). Mass spectrometry conditions were: ESI temperature was set at 500 °C; mass spectrometry ion spray voltage (IS) was 5500 V (positive) and −4500 V (negative); ion source gas I (GSI), gas II (GSII), curtain gas (CUR) was set at 55 psi, 60 psi and 25.0 psi, respectively; and the collision gas (CAD) parameter was high. We obtained QQQ scans as multiple reaction monitoring (MRM) experiments with collision gas (nitrogen) set to 5 psi. In the QQQ, DP and CE for individual MRM transitions were achieved through DP and CE optimization. The resulting data were processed using the mass spectrometry software, Analyst (Version 1.6.3 Applied Biosystems Company, Framingham, MA, USA).

Qualitative analysis was based on the MVDB V2.0 database of Wuhan Maiteville Biotechnology Co., Ltd. (Wuhan, China) and the metabolite information public database. Qualitative analysis of primary and secondary mass spectrometry data was done by referencing existing mass spectrometry databases, such as MassBank, KNAPSACK, HMDB, and METLIN. Finally, the structural analysis of metabolites was also determined. Quantitative analysis: metabolites were quantified via the multiple reaction monitoring mode (MRM) using triple quadrupole mass spectrometry. After obtaining metabolite data from dry-cured beef samples at different periods, the peak area of the mass spectra of all substances was integrated. Further, the mass spectra peaks of the same metabolites in dry-cured beef samples were corrected at the different periods.

#### 2.5.3. Analysis of Metabolite Differences and Metabolic Pathways

The logarithm (log10) of the peak area matrix of dry-cured beef was obtained by eliminating the effect of concentration differences on pattern recognition, and then unit variance scaling was conducted. The MetaboAnalyst 4.0 platform [29] was used to perform principal component analysis (PCA), system clustering and data standardization, pattern recognition, and metabolic network analysis of the dry-cured beef metabolites. All data analyses of this study were based on the self-built MWDB database (Metware Biotechnology Co., Ltd. Wuhan, China). In addition, KEGG pathway analysis was also conducted, and pathways with significantly regulated metabolites were used on metabolite set enrichment analysis to reveal the most relevant pathway for dry-cured beef concerning CG vs. FG and CG vs. RG.

### 2.6. Statistical Analysis

Origin version 2021 (Origin Lab., Hampton, MA, USA) was used to perform statistical analysis and drawing. All data are presented as the mean ± SD (Standard Deviation). Comparisons between groups were conducted using one-way ANOVA or Student’s *t*-test. Moreover, UPLC-MS/MS analyses were performed, where metabolites with a variable important in projection (VIP) ≥1 and fold change (FC) ≥2 or FC ≤0.5 were considered as criteria for screening potential biomarkers. The VIP values were extracted from the orthogonal partial least-squares discriminant analysis (OPLS-DA) results, which also contain score plots and permutation plots. These results were generated using the R package MetaboAnalystR (https://www.metaboanalyst.ca/docs/RTutorial.xhtml, accessed on 21 October 2020). Significantly different metabolites were analyzed based on VIP values and Log_2_FC. A *p*-value < 0.05 was considered statistically significant.

## 3. Results and Discussion

### 3.1. Physicochemical Properties and Protein Hydrolysis and Oxidation Analyses

The results of physicochemical characteristics analyses during the processing of dry-cured beef are shown in Table 1. Moisture was significantly reduced from 74.71% in CK to 25.43% in CS, in agreement with the research results of Ma et al. [30]. The reason may be explained as the salt infiltration and temperature rise, the osmotic pressure of cells increased, and the drip loss was serious after rupture, resulting in a rapid decline in water content. Moreover, moisture can not only effectively influence the stability of products, but also has a positive effect on taste and flavor [11].

The pH value is an important index that affects the color, taste, and shelf life of meat products, and has a significant impact on the quality of meat products. The pH value of dry-cured beef increased from 5.83 in CG to 6.10 in RG, which was caused by the increase of the processing temperature, and the protein may generate basic amino acids, volatile base nitrogen, ammonia, amine, and other basic substances under the action of protease [31]. Dry-cured beef was in a slightly acidic environment (pH value is 5.83~6.10), which could make glutamic acid and sodium glutamate and other umami substances exist at the maximum amount [30], thus ensuring the quality of dry-cured beef and making it have good taste.

Sodium chloride (NaCl) has positive effects on the color, lipid oxidation, and flavor generation of the products. The NaCl content of dry-cured beef showed a significant increasing trend during the processing (*p* < 0.05). It reached 13.56% in the final product, possibly because the addition of NaCl, water loss, and the increase in temperature with the NaCl infiltration resulted in increasing NaCl content during processing. NaCl was added as an ingredient not only to provide saltiness, but also to improve the release of glutamate. It was directly expressed in meat taste substances [32]. Meanwhile, NaCl content has different degrees of influence on the quality of meat products.

The total nitrogen content, non-protein nitrogen content, and PI increased significantly during the processing of dry-cured beef (*p* < 0.05). At the end of the ripening (RG), the above values were 10.42, 1.81, and 17.34, respectively. This was mainly because protein generated peptides, free amino acids, and other substances under the action of enzymes, which had a positive effect on the taste of dry-cured beef [33]. Zhou et al. [34] reported that the proteolytic index of high-quality Jinhua ham was between 14% and 20%, and the value measured in this experiment was within this range, indicating that dry-cured beef had strong protein hydrolysis activity.

The degree of protein oxidation and degradation is a key factor affecting the quality of dry cured meat products. The carbonyl content gradually increased during processing (*p* < 0.05). The concentration of carbonyl in MP is about two times higher than SP. By the end of processing, these values increased by 53.51% and 36.83% (Table 1), respectively. The reason might be the amino acids with the NH^−^ or NH_2_^−^ part on the side chain of the raw protein reacted with the oxidative free radicals generated by lipolysis to convert to carbonyl, and under the action of metal-catalyzed oxidation, α-amidation, and other factors generated carbonyl derivatives [35,36].

The oxidation of the protein leads to the reduction of thiol groups. The prolonged process significantly decreased (*p* < 0.05) the sulfhydryl content of sarcoplasmic protein and myofibril protein in dry-cured beef. The content in the RG decreased by 40.70% and 49.27%, respectively compared with the CG (Table 1). The decrease in sulfhydryl content could be due to the destruction of protein space structure by oxidation, which converted sulfhydryl into intermolecular or intramolecular disulfide bonds or oxidized to sulfinic acid and sulfonic acid [27].

### 3.2. Sensory Assessment

**Figure 1:**
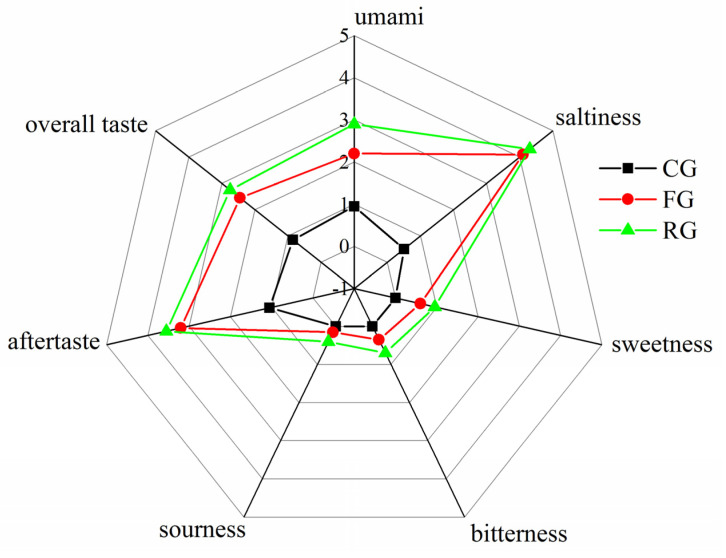
Taste performance of different period for dry-cured beef CG (control group), FG (fermented group), and RG (ripened group).

In this study, the taste performance of dry-cured beef samples from the CG, FG, and RG was analyzed after sensory assessment (Figure 1). Sensory score results indicated that the RG had higher levels of saltiness, umami, after-taste, and overall taste. Umami taste increased significantly (*p* < 0.05), where the sensory score of umami in RG had increased by 67.24% compared with CG. The increase in the sensory score of umami was associated with umami compounds (glutamic acid and aspartic acid) generated during processing [37]. In addition, the saltiness score increased significantly from CG to FG (*p* < 0.05). This could be attributed to the increase in temperature and humidity which promoted salt-penetration into dry-cured beef. It was evident that the saltiness score slowly increased from FG to RG as a result of salt saturation. The results of the after-taste and overall taste showed a significantly increasing trend (*p* < 0.05). It is worth noting that the after-taste has a great influence on the overall taste score [20], which indicated that the aftertaste and overall taste changes could be associated with the generated content of taste substances, such as free amino acids, organic acids, and nucleic acids. Moreover, the sweetness and bitterness scores increased slowly, which can be attributed to the content of sweet amino acids (valine, alanine, and threonine) and bitter substances (isoleucine, leucine, and histidine) produced during the FG and RG process [38]. On the other hand, the low sourness score could have been caused by the low content of sour compounds caused by the addition of salt during processing to inhibit the action of microorganisms [39].

### 3.3. Metabolite Profiles of Dry-Cured Beef

**Figure 2:**
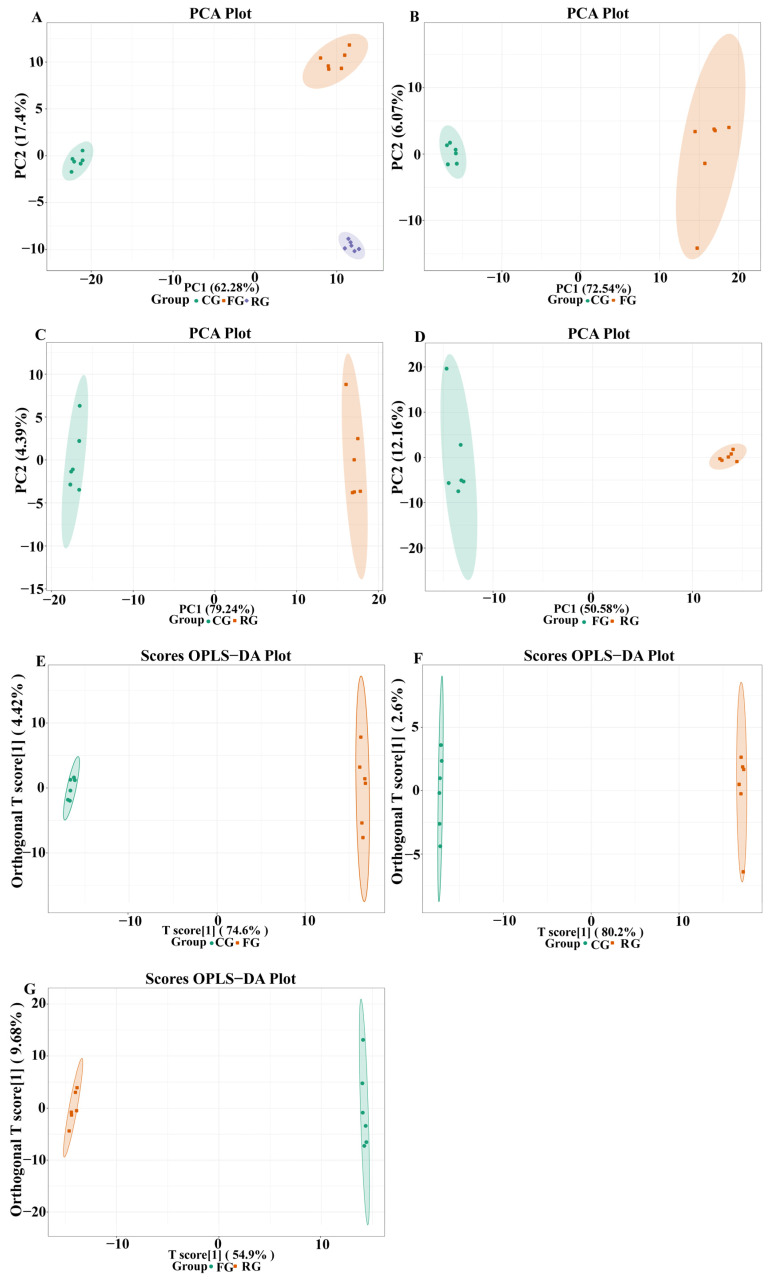
PCA plots showing the different metabolic patterns of dry-cured beef meat. (**A**) All dry-cured beef meat samples, (**B**) meat samples from the CG (control group) and FG (fermented group), (**C**) meat samples from the CG and RG (ripened group), and (**D**) meat samples from the FG and RG. OPLS-DA plots showing clear metabolic differences between dry-cured beef meat samples. (**E**) The CG and FG, (**F**) the CG and RG, and (**G**) the FG and RG. All with 95% confidence.

A total of 400 metabolites were identified from CG, FG, and RG of dry-cured beef using the widely targeted HPLC-MS/MS approach and Maiwei database (Table S1). Principal component analysis (PCA) determined whether there were any global differences between the metabolic profiles of the three-period groups. It was found that 79.68% of the total variation of CG, FG, and RG groups was explained by 62.28% of PC1 and 17.4% of PC2 in the plot, which were well-separated (Figure 2). In addition, the replicated samples were clustered together, indicating that the experiment was reproducible and reliable. A further comparison of CG and FG, CG and RG, and FG and RG indicated that they were separated, with significant differences in metabolite content among the different groups.

The OPLS-DA is an effective multivariate statistical method for screening differential metabolites because it can maximize the differences between groups. The Q^2^ is a critical indicator for evaluating the OPLS-DA model, with a score greater than 0.9 indicating a good model. The R^2^X and Q^2^ values of CG-FG, CG-RG, and FG-RG in OPLS-DA plots were 0.791 and 0.997, 0.828 and 0.998, and 0.645 and 0.994, respectively (Figure 2E–G). Moreover, the Q^2^ values of all groups surpassed 0.9, indicating that the model was highly reliable and had good predictive ability. It can therefore be used for further screening of differential metabolites of dry-cured beef.

### 3.4. Identification of Differential Metabolites

The VIP and FC were used to identify and screen the different metabolites of dry-cured beef in each comparison group. Results of the screening were based on the combined selection criteria of VIP > 1, FC ≥ 2 or ≤0.5, and *p*-value < 0.05. If the difference fold change was ≥2 and VIP > 1, it would mean that the content of the metabolite in the experimental group was two times or more of that of the control group, then the metabolite was defined as an up-regulated metabolite; if the difference fold change was ≤0.5 and VIP > 1, it would mean that the content of the metabolite in the control group was two times or more than that in the experimental group, and the metabolite was defined as a down-regulated metabolite.

**Figure 3:**
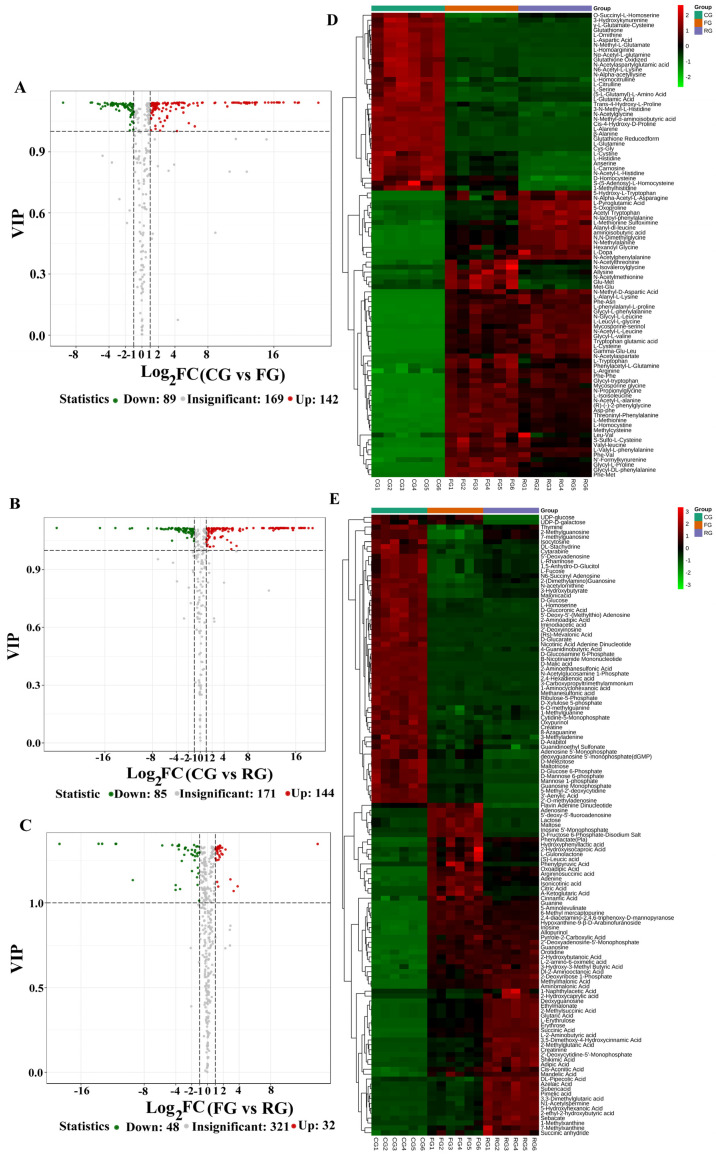
Volcano plots showing the distribution of all metabolites based on their fold-change values (*x*-axis, on a logarithmic scale) and VIP scores (VIP > 1 and VIP < 1). A comparison is made between (**A**) the CG (control group) and FG (fermented group), (**B**) the CG and RG (ripened group) and (**C**) the FG and RG. Up-regulated, down-regulated, and non-differentially expressed metabolites are colored in red, green, and gray, respectively. A clustered heat map based on the differential metabolites of amino acid and small peptides (**D**), and organic acids, nucleotides and carbohydrates (**E**), which shows clear metabolic differences between the RG, FG, and CG. The name of each differential metabolite is provided in the list to the right of the heat map.

The volcano plots (Figure 3) indicate the expression level of differential metabolites. The results of this study showed that there were 231 differentially expressed metabolites (89 down-regulated and 142 up-regulated) between CG and FG, 229 between CG and RG (85 down-regulated and 144 up-regulated), and 80 between FG and RG (48 down-regulated and 32 up-regulated). Furthermore, the differential expressed metabolites of CG, FG, and RG groups were analyzed using a clustering heat map. Results of the differential metabolites of clustered amino acids and small peptides are shown in Figure 3D. The content of 3-N-methyl-L-histidine, cis-4-hydroxy-D-proline, L-alanine, β-alanine, L-glutamine, and cysteinyli-glycine was higher in the CG group than in FG and RG groups. The components of FG and RG were similar, but the content of L-glycine-L-amphetaminoacetic acid, threoninyl-phenylalanine, L-methionine, L-homocystine, S-methyl-L-cysteine, glycyl-L-proline, glycyl-DL-phenylalanine and L-phenylalanyl-L-methionine was higher in the FG. However, RG contained higher levels of alanyl-dl-leucine, aminoisobutyric acid, N, N-dimethylglycine, N-methylalanine, hexanoyl glycine, and L-dopa. Figure 3E presents the results of differential metabolites of organic acids, nucleotides, and carbohydrates. Moreover, 4-guanidinobutyric acid, D-(+)-malic acid, taurine of organic acids, and its derivatives, β-nicotinamide mononucleotide of nucleotides, and its metabolites, D-glucosamine 6-phosphate, and N-acetylglucosamine 1-phosphate of carbohydrates, and its metabolites were higher in the CG than in FG and RG. The adenosine, inosine 5′-monophosphate of nucleotides, and their metabolites and lactose, maltose, D-fructose, and 6-phosphate-disodium salt of carbohydrates and their metabolites were significantly higher in the FG than in CG and RG. Finally, organic acids and its derivatives included ethylmalonic acid, methylsuccinic acid, glutaric acid, succinic acid, L-2-aminobutyric acid, 2-methylglutaric acid, DL-pipecolic acid, azelaic acid, subericacid, pimelic acid, 3,3-dimethylglutaric acid, N-acetylspermine, 5-hydroxyhexanoic acid, 2-ethyl-2-hydroxybutyric acid, nucleotides, and their metabolites 2′-deoxyguanosine, and L-erythrulose, and erythrose of carbohydrates, and its metabolites were prominently higher in the RG than in CG and FG.

### 3.5. Changes in Metabolites Composition of Dry-Cured Beef

**Figure 4:**
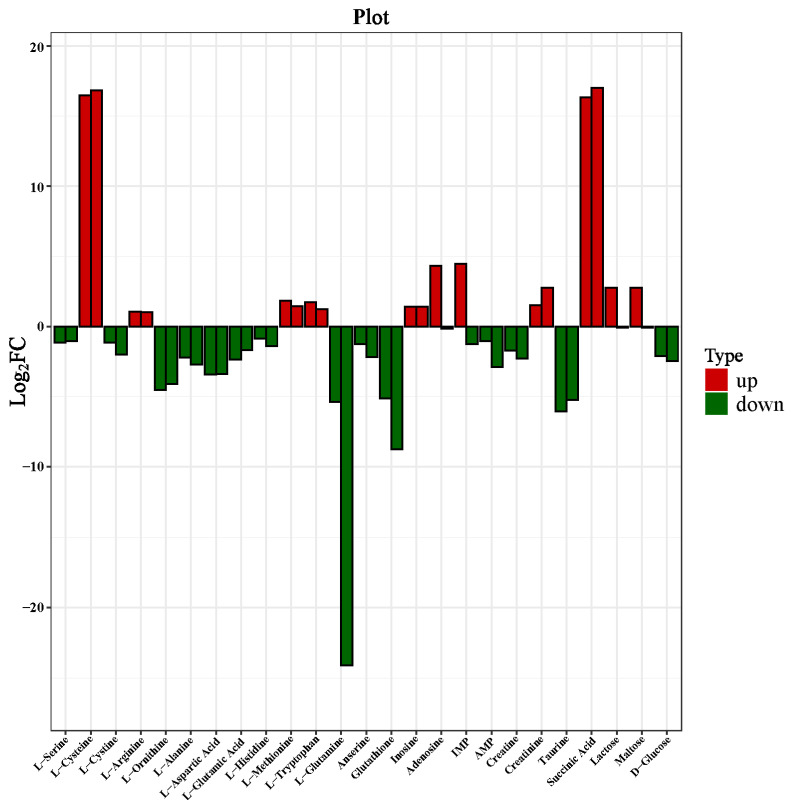
The relative content of different metabolites of dry-cured beef at the processing stage of FG (fermented group) and RG (ripened group), with CG (control group) used as a control.

The relative content of the 25 most significant differentially expressed metabolites was analyzed at a FG and RG ratio, with CG as a control, to fully understand the dynamics of these metabolites. Figure 4 shows the relative content of amino acids, small peptides, organic acids, nucleotides, and sugars.

Results of this study indicated that the amino acids and small peptide metabolites of dry-cured beef were L-serine, L-cysteine, L-cystine, L-arginine, L-alanine, L-aspartic acid, L-glutamic acid, L-methionine, L-histidine, L-tryptophan, L-glutamine, anserine, and glutathione. A previous study reported that most amino acids are formed by the hydrolysis of proteins and peptides under the action of enzymes [40], as a result of the long processing time. Another study reported that amino acids were used as the precursors of flavor compounds in dry-cured beef [14], among which L-aspartic acid, L-glutamic acid, and L-glutamine are associated with an umami taste, L-arginine, L-histidine, and L-methionine contributed to bitter taste, and L-serine, L-alanine, L-cysteine and L-cystine led to a sweet taste. Notably, L-glutamine provides a typical umami taste that cannot be replaced by other tastes [19]. Moreover, L-cysteine determines meat flavor volatiles. In this study, L-cysteine was significantly up-regulated in FG and RG, which can be attributed to the conversion of ribose-5-phosphate to cysteine due to the hydrolysis of proteins during processing. L-glutamine was significantly down-regulated in FG and RG compared to the CG because glutamine produced glutamate under the action of glutaminase. Meanwhile, 2-oxoglutarate was produced under the action of glutaminase II, and then glutamate was generated under the action of glutamate synthase. Glutathione is an important meaty precursor which can improve food taste and increase nutritional value. In this study, glutathione was significantly down-regulated in the two groups compared to CG because it was catalyzed by glutathione hydrolase to produce L-cysteinyl-glycine and L-glutamate, and its interaction with the Maillard reaction had a greater contribution to the formation of a meaty taste. Furthermore, anserine was less down-regulated in FG and RG compared to the CG. Another study reported that it has antioxidant and buffering properties, and that it can improve the taste when the pH is greater than 6 [41]. Compared with CG, low up-regulation was observed in L-arginine, L-methionine, and L-tryptophan content, while less down-regulation was observed in L-serine, L-cystine, L-glutamic acid, and L-histidine content. Liu et al. [42] speculated that the taste characteristics of non-volatile components in Yunnan dry-cured ham were closely associated with their content, and found that histidine, alanine, and aspartic acid also had an auxiliary effect on the taste. Therefore, the interaction between free amino acids and small peptides contributed to the formation of a unique flavor in dry-cured beef.

Organic acids are an important part of taste compounds. The results of this study indicated that creatine, creatinine, taurine, and succinic acid were the main differentially expressed organic acids in dry-cured beef. Succinic acid showed a significant up-regulation trend in FG and RG compared to the CG, which can be attributed to the catalysis of 2-oxoglutarate by glutarate dioxygenase to produce succinic acid. Succinate provides a sour taste as a result of the reaction of succinic acid, which can increase the internal redness of cooked beef [43]. On the other hand, taurine, which is one of the primary bioactive substances to human health, was significantly down-regulated. Furthermore, this continuous loss could have been caused by salting and conversion into other substances [44]. Creatine is a valuable metabolite and an important component associated with energy transfer and ATP formation [45]. Results of this study indicated that creatine was less down-regulated in FG and RG compared to the CG, which could be catalyzed by creatine kinase to creatine phosphate, and the content can be attributed to the type of metabolism [46]. Creatine has been identified as the taste-active compound in duck meat [13], and has caused the bitterness and sweetness of dry-cured ham [20]. Creatinine, which is a product of the creatine phosphate metabolism, is a compound with taste activity and is the main cause of a bitter taste. It was shown that it was less up-regulated in FG and RG compared to CG, which was converted from creatine and creatine phosphate at a relatively low rate [19]. According to Parket et al. [46], it is evident that a combination of creatinine and creatine produces a synergistic effect.

Nucleic acids and their derivatives are mainly produced by the metabolism of nucleotides, which are mainly derived from the metabolism of adenosine triphosphate (ATP). Further, ATP is degraded to adenosine diphosphate (ADP) and reduced to adenosine monophosphate (AMP), followed by reduction to disodium Inosine-5′-Monophosphate (IMP). Subsequently, inosine (HxR) and hypoxanthine (Hx) are produced [41,47]. In this study, adenosine, HxR, IMP, and AMP were significantly differentially expressed metabolites of dry-cured beef. Particularly, adenosine and IMP were significantly up-regulated in the FG. However, adenosine was the least down-regulated metabolite in the RG, which can be attributed to the relative content of nucleotide degradation products, such as AMP. IMP, formed by HxR and phosphoric acid, is a flavor enhancer and can also perceive umami taste [48]. On the other hand, adenosine is an important intermediate for the synthesis of ATP, adenine, AMP, and adenosine arabinoside, as well as an inhibitory neurotransmitter. In a previous study, adenosine degraded AMP to provide an umami taste [42]. It was reported that AMP was significantly down-regulated in FG and RG since the generated adenosine was involved in the synthesis of AMP, under the action of 5′-nucleotidase and nucleosides. Moreover, there was less up-regulation of HxR, which was formed by the combination of hypoxanthine and a ribose ring. The AMP and HxR are closely associated with energy and nucleic acid metabolism, which contributed to umami and bitter tastes, respectively [49,50]. Therefore, these findings suggest that nucleic acids and their derivatives play an auxiliary role in producing the umami taste of dry-cured beef.

The main significant metabolites of carbohydrates in dry-cured beef are D-glucose, lactose, and maltose. The results of the current study indicate a significant decrease in D-glucose in FG and RG compared to the CG, which might be due to the Maillard reaction between free amino acids and glucose in dry-cured beef. Glucose is produced by degradation of glycogen and is converted to pyruvate through glycolysis. Subsequently, pyruvate is converted into other metabolites through various metabolic pathways [51]. It is worth noting that glucose is a source of sweetness. Thus, free glucose, as previously observed by Kang et al. [52] in Yunnan dry-cured hams, can be more conducive to taste formation. In addition, our results showed that lactose was significantly up-regulated in the FG and down-regulated in the RG, which could be due to the metabolism of lactose to produce lactic acid and its reaction with other substances, thereby affecting the meat quality and pH of dry-cured beef [42]. Maltose is the product of glycogen breakdown, and it provides a unique taste to dry-cured beef. In this study, the level of maltose was high in the FG and low in the RG, as a result of maltose catalysis by 4-α-glucanotransferase to generate D-glucose. Therefore, we postulate that it provided sweetness to the final product.

Notably, amino acids, small peptides, organic acids, nucleotides, and carbohydrates interacted with each other with a synergistic or suppressive effect. Therefore, the concordance of various metabolite components contributed to the overall taste and flavor of dry-cured beef.

### 3.6. Pathway Analysis

**Figure 5:**
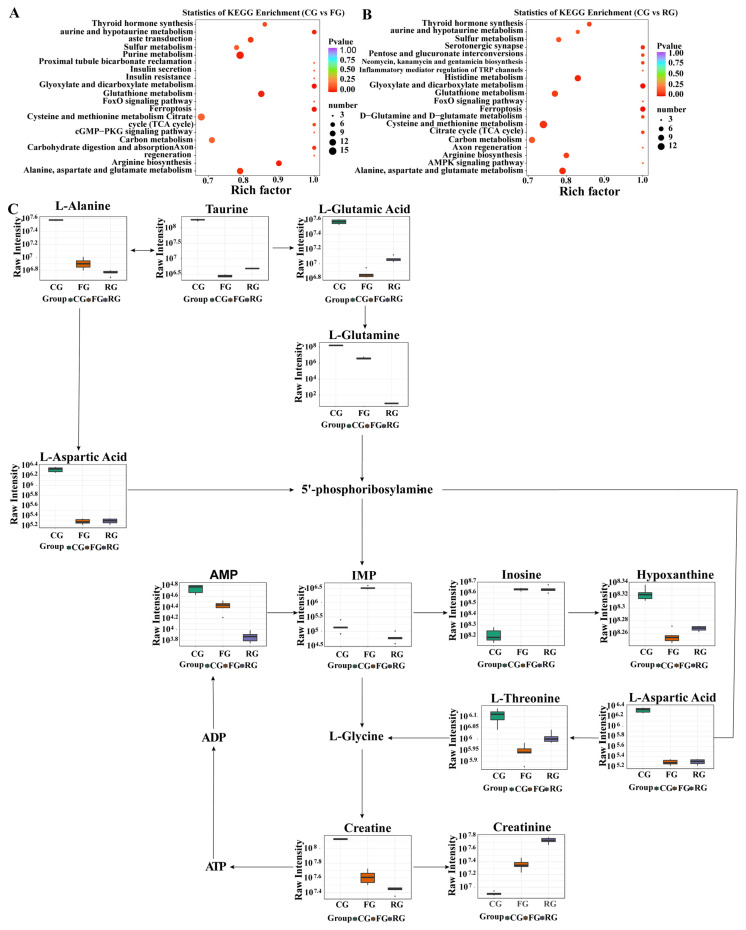
Dry-cured beef metabolic pathway analysis based on the significantly differentially expressed metabolites in water-soluble compounds in CG—control group versus FG—fermented group (**A**) and CG versus RG—ripened group (**B**). Degree of enrichment was analyzed using a rich factor, *p*-value, and the number of dry-cured beef metabolites were enriched in each pathway. The size of the bubble indicates the amount of significantly differential metabolites in water-soluble compounds which were enriched in this pathway, and the point with different gradation of color represents the scope of the *p*-value. The higher value of the rich factor represents the higher degree of enrichment, while the lower *p*-value represents the more significant degree of enrichment. The umami taste of dry-cured beef was produced during processing (**C**). The violin plot illustrates the metabolites found in the dry-cured beef meat. Metabolites in black font were not detected.

Metabolic pathway analysis is a useful method for directly analyzing the internal connections of metabolites, which can reconstruct the biochemical reaction network [53]. The main pathways of dry-cured beef metabolism are summarized and outlined in Figure 5. Results of this study showed that dry-cured beef in different periods reacted to changes in the metabolites, and then affected the taste of meat through five related metabolic pathways. These five pathways included the glyoxylate and dicarboxylate metabolism, glutathione metabolism, alanine, aspartate and glutamate metabolism, and arginine biosynthesis, as well as the taurine and hypotaurine metabolism [5,54].

Umami is a very important component for the taste of dry-cured meat products, mainly obtained from nucleotides after degradation of ATP to ADP and subsequent reduction to AMP and then IMP [47]. It can also be provided by conversion of taurine to 2-oxoglutarate, followed by the formation of glutamic acid under the action of glutamate synthase, and subsequently, glutamine under the catalysis of glutamine synthetase [55]. Meanwhile, taurine is catalyzed by taurine-pyruvate aminotransferase to produce alanine, while N-carbamoyl-L-aspartate generates aspartic acid under the action of aspartate carbamoyltransferase, followed by conversion into alanine [56]. Previous studies have shown that the important components of umami taste are nucleotides [28] and amino acids [14]. Figure 5C shows that most metabolites are the ones commonly reported as associated with an umami taste; in particular, AMP [42], IMP [48], glutamic acid and aspartic acid [14] are known to cause an umami taste. The IMP, which is an intermediate metabolite of taurine and hypotaurine metabolism, alanine, aspartic acid, glutamate metabolism, and the purine metabolic pathway, is generated from glutamic acid and aspartic acid. It is considered one of the major determinants of the umami taste in meat [28]. IMP is mainly involved in the reaction of HxR, Hx, threonine, creatine, and creatinine. Previous studies have reported that threonine and IMP exhibit umami synergy [57], whereas HxR and glutamate enhance the umami taste [11]. Furthermore, creatine can affect meat tenderness, water retention, and the amino acid content of meat [5]. Our findings suggest that alanine, aspartate, and glutamate metabolism, purine metabolism, and taurine and hypotaurine metabolism are important metabolic pathways which affect the umami characteristics of dry-cured beef.

### 3.7. Analysis of the Correlation between Sensory Assessment and Taste Metabolites

**Figure 6:**
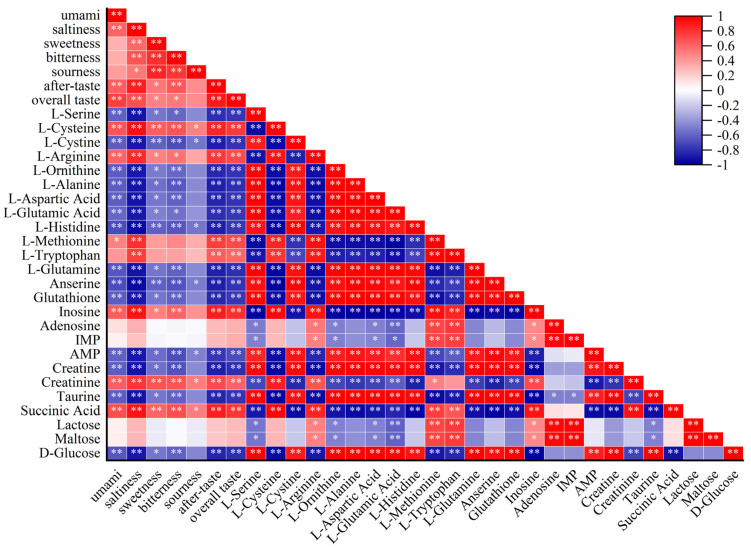
Correlation between sensory assessment and metabolites in dry-cured beef.

The correlation analysis between sensory assessment and characteristic taste metabolites in dry-cured beef during processing is shown in Figure 6. The color depth is remarkably related to the absolute value of the correlation coefficient. There is a strong correlation between sensory evaluation and taste metabolites in dry-cured beef. Umami has a significant positive correlation with saltiness, aftertaste, overall taste, L-cysteine, L-arginine, inosine, creatinine, and succinic acid. Additionally, L-serine, L-cystine, L-ornithine, L-alanine, L-aspartic acid, L-histidine, L-glutamine, anserine, glutathione, AMP, creatine, taurine, and D-glucose showed a significant negative correlation, which might be due to the conversion into other umami substances, as well as other substances [5,41]. L-glutamic acid is significantly positively correlated with L-glutamine, anserine, glutathione, adenylate, taurine, and D-glucose. L-aspartic acid is significantly positively correlated with L-glutamine, anserine, glutathione, and AMP. AMP is significantly positively correlated with L-aspartic acid, L-glutamic acid, L-glutamine, and glutathione. It mainly came from free amino acids, small peptides by proteolysis [3], nucleotide degradation compounds [47], organic acids, carbohydrates, which play an important role in the formation of the sensory quality and overall taste of dry-cured beef [58]. Therefore, the formation of the taste and flavor of dry-cured beef is closely related to the interaction between different metabolites during processing.

## 4. Conclusions

This study explored the sensory properties and differential metabolites influencing the taste of dry-cured beef during fermentation and maturity. Clearly, it was crucial to establish the differences in the metabolic composition profiles of dry-cured beef. The overall sensory score is higher in the final product with salty and umami tastes. Amino acid and small peptides could be the main source of the taste in the dry-cured beef during processing. Moreover, organic acids, nucleotides (and their metabolites), and carbohydrates affected the taste. The metabolites that influenced the umami taste of dry-cured beef were associated with alanine, aspartate, glutamate, purine, taurine, and hypotaurine metabolism. Correlation analysis revealed that the formation of the sensory quality and overall taste of dry-cured beef is collectively influenced by the joint effect of metabolites. The present study gives us new insight to understand the relationship between the metabolic changes and taste of dry-cured beef. These data can be used to develop strategies for improving the processing of dry-cured beef.

## Figures and Tables

**Table 1:** Analysis of physicochemical characteristics of dry-cured beef during processing.

	CG	FG	RG
Moisture (%)	74.71 ± 0.68 ^aA^	34.93 ± 0.42 ^bB^	25.43 ± 0.22 ^cC^
pH value	5.83 ± 0.03 ^cC^	5.91 ± 0.01 ^bB^	6.1 ± 0.02 ^aA^
NaCl (%)	0.05 ± 0.01 ^cC^	10.36 ± 0.28 ^bB^	13.56 ± 0.34 ^aA^
Total nitrogen (%)	8.98 ± 0.40 ^cC^	9.23 ± 0.52 ^bB^	10.42 ± 0.49 ^aA^
Non-protein nitrogen (%)	0.67 ± 0.64 ^cC^	1.18 ± 0.35 ^bB^	1.81 ± 0.18 ^aA^
Proteolysis index (%)	7.46 ± 0.48 ^cC^	12.82 ± 0.32 ^bB^	17.34 ± 0.15 ^aA^
SP Carbonyl content (nmol/mg)	5.36 ± 0.01 ^cC^	8.52 ± 0.11 ^bB^	11.53 ± 0.18 ^aA^
MP Carbonyl content (nmol/mg)	11.56 ± 0.07 ^cC^	14.97 ± 0.16 ^bB^	18.3 ± 0.13 ^aA^
SP Sulfhydryl content (nmol/mg)	41.05 ± 0.51 ^aA^	28.43 ± 0.26 ^bB^	24.34 ± 0.37 ^cC^
MP Sulfhydryl content (nmol/mg)	32.13 ± 0.45 ^aA^	19.71 ± 0.22 ^bB^	16.3 ± 0.31 ^cC^

Note: Different lowercase letters in the same row indicate significant differences *p* < 0.05, and different uppercase letters indicate highly significant differences *p* < 0.01. Sodium chloride (NaCl), Myofibrin (MP) and sarcoplasmic protein (SP), CG (control group), FG (fermented group), and RG (ripened group).

## Data Availability

The data presented in this study are available on request from the corresponding author. The data are not publicly available due to institutional privacy.

## Supplementary Materials

The following supporting information can be downloaded at: www.mdpi.com/article/10.3390/foods11040531/s1, Table S1: Metabolites detected in dry-cured beef during each processing stage.

## References

[1] Sha K., Lang Y.M., Sun B.Z., Su H.W., Li H.P., Zhang L., Lei Y.H., Li H.B., Zhang Y. (2017). Changes in lipid oxidation, fatty acid profile and volatile compounds of traditional kazakh dry-cured beef during processing and storage. J. Food Process. Preserv..

[2] Zhou C.Y., Bai Y., Wang C., Li C.B., Xu X.L., Pan D.D., Cao J.X., Zhou G.H. (2021). 1H NMR-based metabolomics and sensory evaluation characterize taste substances of Jinhua ham with traditional and modern processing procedures. Food Control.

[3] Suwandy V., Carne A., Remy V.D.V., Bekhit E.D.A., Hopkins D.L. (2015). Effect of pulsed electric field on the proteolysis of cold boned beef *M. Longissimus lumborum* and *M. Semimembranosus*. Meat Sci..

[4] Feng L., Qiao Y., Zou Y.F., Huang M., Kang Z.L., Zhou G.G. (2014). Effect of Flavourzyme on proteolysis, antioxidant capacity and sensory attributes of Chinese sausage. Meat Sci..

[5] Xiao Z.C., Ge C.G., Zhou G.H., Zhang W.G., Liao G.Z. (2019). H NMR-based metabolic characterization of Chinese Wuding chicken meat. Food Chem..

[6] Tian X., Li Z.J., Chao Y.Z., Wu Z.Q., Zhou M.X., Xiao S.T., Zeng J., Zhe J. (2020). Evaluation by electronic tongue and headspace-GC-IMS analyses of the flavor compounds in dry-cured pork with different salt content. Food Res. Int..

[7] Martín-Gómez A., Segura-Borrego M.P., Ríos-Reina R., Cardador M.J., Callejón R.M., Morales M.L., Rodríguez-Estévezd V., Arce L. (2022). Discrimination of defective dry-cured Iberian ham determining volatile compounds by non-destructive sampling and gas chromatography. LWT-Food Sci. Technol..

[8] Karpiński P., Kruszewski B., Stachelska M.A., Szabowska E. (2020). Development of volatiles profile of Kumpiak podlaski dry-cured ham during traditional ripening. Int. J. Food Sci. Technol..

[9] Koutsidis G., Elmore J.S., Oruna-Concha M.J., Campo M.M., Wood J.D., Mottram D.S. (2008). Water-soluble precursors of beef flavour: I. Effect of diet and breed. Meat Sci..

[10] Graham S.F., Kennedy T., Chevallier O., Gordon A., Farmer L., Elliott C., Moss B. (2010). The application of NMR to study changes in polar metabolite concentrations in beef longissimus dorsi stored for different periods post mortem. Metabolomics.

[11] Sugimoto M., Sugawara T., Obiya S., Enomoto A., Kaneko M., Ota S., Soga T., Tomita M. (2020). Sensory properties and metabolomic profiles of dry-cured ham during the ripening process. Food Res. Int..

[12] Koutsidis G., Elmore J.S., Oruna-Concha M.J., Campo M.M., Wood J.D., Mottram D.S. (2008). Water-soluble precursors of beef flavour. Part II: Effect of post-mortem conditioning. Meat Sci..

[13] Lou X.W., Ye Y.F., Wang Y., Sun Y.Y., Pan D.D., Cao J.X. (2018). Effect of high-pressure treatment on taste and metabolite profiles of ducks with two different vinasse-curing processes. Food Res. Int..

[14] Zhou C.Y., Wang Y., Cao J.X., Chen Y.J., Liu Y., Sun Y.Y., Pan D.D., Ou C.R. (2016). The effect of dry-cured salt contents on accumulation of non-volatile compounds during dry-cured goose processing. Poult. Sci..

[15] Fang Z.Z., Gonzalez F.J. (2014). LC–MS-based metabolomics: An update. Arch. Toxicol..

[16] Dunn W.B., Broadhurst D.I., Helen J., Atherton H.J., Goodacre R., Griffin J.L. (2011). Systems level studies of mammalian metabolomes: The roles of mass spectrometry and nuclear magnetic resonance spectroscopy. Chem. Soc. Rev..

[17] Liao R.y., Xia Q., Zhou C.Y., Geng F., Wang Y., Sun Y.Y., He J., Pan D.D., Cao J.X. (2021). LC-MS/MS-based metabolomics and sensory evaluation characterize metabolites and texture of normal and spoiled dry-cured hams. Food Chem..

[18] Mabuchi R., Ishimaru A., Tanaka M., Kawaguchi O., Tanimoto S. (2019). Metabolic profiling of fish meat by GC-MS analysis, and correlations with taste attributes obtained using an electronic tongue. Metabolites.

[19] Zhang J., Yi Y., Pan D.D., Zhou G.H., Wang Y., Dang Y.L., He J., Li G., Cao J.X. (2019). H1-NMR-based metabolomics profiling and taste of boneless dry-cured hams during processing. Food Res. Int..

[20] Zhang J., Ye Y.F., Sun Y.Y., Pan D.D., Ou C.G., Dang Y.L., Wang Y., Cao J.X., Wang D.Y. (2018). H1-NMR and multivariate data analysis of the differences of metabolites in five types of dry-cured hams. Food Res. Int..

[21] Liu S.X., Zhang Y.W., Zhou G.H., Bao Y.J., Ren X.P., Zhu Y.X., Peng Z.Q. (2019). Protein degradation, color and textural properties of low sodium dry cured beef. Int. J. Food Prop..

[22] Shi S., Kong B.H., Wang Y., Liu Q., Xia X.F. (2020). Comparison of the quality of beef jerky processed by traditional and modern drying methods from different districts in Inner Mongolia. Meat Sci..

[23] AOAC (1995). Official Methods of Analysis of AOAC International.

[24] Careri M., Mangia A., Barbieri G., Bouoni L., Virgili R., Parolari G. (1993). Sensory property relationships to chemical data of italian-type dry-cured ham. J. Food Sci..

[25] Ran L.D., Li W.H., Zhao C., Zhong Y.Y., Yuan H.C., Yan Q.Q., Zhu W.C., Dong J. (2021). Effect of Tea Polyphenol/Hydroxypropyl-β-Cyclodextrin Inclusion Complex on Myofibrillar Protein Oxidation in Ovine Tripe during Refrigerated Storage. Food Sci..

[26] Zhang B., Fang C.D., Hao G.J., Zhang Y.Y. (2018). Effect of kappa-carrageenan oligosaccharides on myofibrillar protein oxidation in peeled shrimp (*Litopenaeus vannamei*) during long-term frozen storage. Food Chem..

[27] Jia D., You J., Hu Y., Liu R., Xiong S. (2015). Effect of CaCl_2_ on denaturation and aggregation of silver carp myosin during setting. Food Chem..

[28] Yang Y., Wang Y., Pan D.D., Zhang Y., He J., Xia Q., Cao J.X. (2020). The application of H NMR to explore the taste difference caused by taste-active metabolites of different Chinese sauce-stewed beef. Food Sci. Nutr..

[29] Xia J.G., Sinelnikov I.V., Han B., Wishart D.S. (2015). MetaboAnalyst 3.0—Making metabolomics more meaningful. Nucleic Acids Res..

[30] Ma Y.M., Lu S.L., Wang Q.L. (2016). Study on the variation of physicochemical properties during dry-cured mutton ham processing. Food Ind..

[31] Zhou C.Y., Pan D.D., Bai Y., Li C.B., Xu X.L., Zhou G.H., Cao J.X. (2019). Evaluating endogenous protease of salting exudates during the salting process of Jinhua ham. LWT-Food Sci. Technol..

[32] Martuscelli M., Lupieri L., Chaves-Lopez C., Mastrocola D., Pittia P. (2015). Technological approach to reduce NaCl content of traditional smoked dry-cured hams: Effect on quality properties and stability. J. Food Sci. Technol..

[33] Ma Y.M., Lu S.L., Li K.X., Jiang C.H., Peng X.L., Pei L.Y., Wang Y.H., Chu X.H. (2014). Study on proteolysis during processing of dry-cured mutton ham. Food Ferment. Ind..

[34] Zhou G.H., Zhao G.M. (2007). Biochemical changes during processing of traditional Jinhua ham. Meat Sci..

[35] Yang J., Xiong Y. (2018). Comparative time-course of lipid and myofibrillar protein oxidation in different biphasic systems under hydroxyl radical stress. Food Chem..

[36] Saiga A., Tanabe S., Nishimura T. (2003). Antioxidant activity of peptides obtained from porcine myofibrillar proteins by protease treatment. J. Agric. Food Chem..

[37] Sforza S., Galaverna G., Schivazappa C., Marchelli R., Dossena A., Virgili R. (2006). Effect of extended aging of Parma dry-cured ham on the content of oligopeptides and free amino acids. J. Agric. Food Chem..

[38] Zhao G.M., Zhou G.H., Tian W., Xu X.L., Wang Y.L., Luo X. (2005). Changes of alanyl aminopeptidase activity and free amino acid contents in biceps femoris during processing of Jinhua ham. Meat Sci..

[39] Dang Y.L., Wang Z., Xu S.Y. (2008). Methods for extracting the taste compounds from water soluble extract of Jinhua ham. Eur. Food Res. Technol..

[40] Feng X., Hang S.S., Zhou Y.G., Liu Q., Yang H.S. (2018). Bromelain Kinetics and Mechanism on Myofibril from Golden Pomfret (*Trachinotus blochii*). J. Food Sci..

[41] Zhao X., Wu J., Chen L., Yang H.S. (2019). Effect of vacuum impregnated fish gelatin and grape seed extract on metabolite profiles of tilapia (*Oreochromis niloticus*) fillets during storage. Food Chem..

[42] Liu S.Y., Wang G.Y., Xiao Z.C., Pu Y.H., Ge C.R., Liao G.Z. (2019). 1H-NMR-based water-soluble low molecular weight compound characterization and free fatty acid composition of five kinds of Yunnan dry-cured hams. LWT-Food Sci. Technol..

[43] Ramanathan R., Mancini R.A., Dady G.A., Van B.C.B. (2013). Effects of succinate and pH on cooked beef color. Meat Sci..

[44] Wang Y.Y., Li C.S., Li L.H., Yang X.Q., Chen S.J., Wu Y.Y., Zhao Y.Q., Wang J.X., Wei Y., Yang D.Q. (2019). Application of UHPLC-Q/TOF-MS-based metabolomics in the evaluation of metabolites and taste quality of Chinese fish sauce (yu-lu) during fermentation. Food Chem..

[45] Sundekilde U.K., Rasmussen M.K., Young J.F., Bertram H.C. (2017). High resolution magic angle spinning NMR spectroscopy reveals that pectoralis muscle dystrophy in chicken is associated with reduced muscle content of anserine and carnosine. Food Chem..

[46] Park J.N., Watanabe T., Endoh K.I., Watanabe K., Abe H. (2002). Taste-active components in a Vietnamese fish sauce. Fish. Sci..

[47] Hong H., Regenstein J.M., Luo Y.K. (2017). The importance of ATP-related compounds for the freshness and flavor of post-mortem fish and shellfish muscle: A review. Crit. Rev. Food Sci. Nutr..

[48] Yang Y., Ye Y.F., Pan D.D., Sun Y.Y., Wang Y., Cao J.X. (2018). Metabonomics profiling of marinated meat in soy sauce during processing. J. Sci. Food Agric..

[49] Guo Y.R., Gu S.Q., Wang X.C., Zhuang K.J., Wang S., Shi J. (2015). Nutrients and non-volatile taste compounds in Chinese mitten crab by-products. Fish. Sci..

[50] Zou Y.H., Kang D.C., Liu R., Qi J., Zhou G.H., Zhang W.G. (2018). Effects of ultrasonic assisted cooking on the chemical profiles of taste and flavor of spiced beef. Ultrason. Sonochem..

[51] Chen D.A., Ye Y.F., Chen J.J., Zhan P.P., Lou Y.J. (2017). Molecular nutritional characteristics of vinasse pike eel (*Muraenesox cinereus*) during pickling. Food Chem..

[52] Kang S.M., Kang G.H., Seong P., Kim Y.C., Park B.Y., Cho S.H. (2013). Effect of Cooking Condition on the Water-Soluble Flavor Precursors in Various Beef Muscles from Hanwoo (Korean Cattle). Korean J. Food Sci. Anim. Resour..

[53] Klamt S., Stelling J.B. (2003). Two approaches for metabolic pathway analysis?. Trends Biotechnol..

[54] Cônsolo N.R.B., Rosa A.F., Barbosa L.C.G.S., Maclean P.H., Higuera P.A., Colnago L.A., Titto E.A.L. (2021). Preliminary study on the characterization of *Longissimus lumborum* dark cutting meat in Angus × Nellore crossbreed cattle using NMR-based metabolomics. Meat Sci..

[55] Franciosa I., Ferrocino I., Giordano M., Mounier J., Rantsiou K., Cocolin L. (2021). Specific metagenomic asset drives the spontaneous fermentation of Italian sausages. Food Res. Int..

[56] Huang S. (2017). Integrating Transcriptomics and Metabolomics to Reveal Metabolism of Amino Acid of Streptococcus thermophilus TF96.

[57] Sun H.W., Wang J.Z., Zhang C.H., Li X., Xu X., Dong X.B., Hu L., Li C.H. (2014). Changes of flavor compounds of hydrolyzed chicken bone extracts during Maillard reaction. J. Food Sci..

[58] Yang Y., Pan D.D., Wang Y., He J., Yue Y., Xia Q., Zhou G.H., Cao J.X. (2020). Effect of reconstituted broth on the taste-active metabolites and sensory quality of stewed and roasted pork-hock. Foods.

