# Supplementary material for: Sensory Properties and Main Differential Metabolites Influencing the Taste Quality of Dry-Cured Beef during Processing
Source: Foods. 2022 Feb 12;11(4):531. doi: 10.3390/foods11040531 (PMC8870990; doi:10.3390/foods11040531)
Supplement: Supplementary file 1 [file foods-11-00531-s001.zip › foods-1575251-supplementary.pdf]

Table S1. Metabolites detected in dry-cured beef during each processing stage.

MVDB Index	Compounds	Q1 (Da)	Formula	CK1	CK2	CK3	CK4	CK5	CK6	FJ1	FJ2	FJ3	FJ4	FJ5	FJ6	CS1	CS2	CS3	CS4	CS5	CS6	mix01	mix02	mix03
MEDN0005	L-Threonine	**118.0504432**	C4H9NO3	1,100,000	1,200,000	1,370,000	1,330,000	1,280,000	1,300,000	875,000	754,000	964,000	873,000	917,000	866,000	1,100,000	989,000	965,000	1,030,000	1,010,884	973,000	769,000	963,000	971,000
MEDN0017	L-Pyroglutamic Acid	**128.0347931**	C5H7NO3	555,000	667,000	628,000	575,000	569,000	591,000	1,580,000	1,490,000	1,480,000	1,430,000	1,770,000	1,710,000	4,430,000	4,650,000	4,900,000	4,390,000	4,691,360	5,080,000	2,000,000	2,290,000	2,440,000
MEDN0018	L-Serine	**104.0347931**	C3H7NO3	668,000	756,000	825,000	801,000	716,000	782,000	345,000	304,000	330,000	400,000	342,000	348,000	402,000	363,000	345,000	369,000	366,016	351,000	306,000	342,000	365,000
MEDN0022	(5-L-Glutamyl)-L-Amino Acid	**217.0824716**	C8H14N2O5	3,070,000	4,570,000	4,510,000	4,760,000	3,700,000	4,280,000	301,000	223,000	272,000	198,000	361,000	452,000	829,000	782,000	795,000	804,000	796,336	772,000	986,000	1,300,000	1,350,000
MEDN0028	4-Hydroxy-L-Glutamic Acid	**162.0402724**	C5H9NO5	35,600	33,300	35,300	45,400	30,700	36,000	18,500	9,970	12,300	39,400	12,600	18,200	24,900	39,500	12,300	23,300	25,929	29,600	25,100	13,400	19,100
MEDN0040	Glycyl-L-Proline	**171.0769923**	C7H12N2O3	5,410	10,700	10,100	11,200	9,770	13,500	217,000	186,000	247,000	236,000	228,000	189,000	117,000	165,000	132,000	128,000	134,582	130,000	145,000	140,000	149,000
MEDN0041	Hexanoyl Glycine	**172.0973933**	C8H15NO3	5,660	5,840	7,750	10,400	10,900	8,530	234,000	193,000	384,000	91,400	189,000	311,000	327,000	317,000	357,000	413,000	356,106	367,000	166,000	175,000	184,000
MEDN0045	L-Cysteine	**120.0119495**	C3H7NO2S	9	9	9	9	9	9	810,000	789,000	834,000	720,000	876,000	833,000	929,000	1,070,000	1,130,000	989,000	1,059,088	1,180,000	583,000	733,000	621,000
MEDN0056	N-Acetyl-L-Leucine	**172.0973933**	C8H15NO3	202,000	248,000	256,000	254,000	251,000	226,000	8,540,000	5,930,000	10,700,000	7,660,000	9,760,000	8,670,000	9,700,000	10,200,000	10,800,000	9,530,000	10,210,360	10,800,000	5,110,000	5,960,000	5,960,000
MEDN0061	N-Phenylacetylglycine	**192.0660932**	C10H11NO3	52,600	60,000	61,600	59,200	56,000	59,300	55,900	49,600	60,700	55,100	56,400	57,800	64,200	67,900	64,300	70,000	66,216	64,600	54,700	57,200	64,700
MEDN0062	N-Propionylglycine	**130.0504432**	C5H9NO3	781,000	883,000	909,000	843,000	884,000	909,000	2,660,000	2,450,000	2,810,000	2,620,000	2,640,000	2,790,000	2,210,000	2,180,000	2,150,000	2,350,000	2,223,960	2,230,000	1,690,000	2,150,000	2,260,000

MEDN0065	O-Phospho-L-Serine	**184.0011236**	C3H8NO6P	70,600	64,200	64,500	88,900	86,000	91,200	112,000	98,500	141,000	100,000	96,300	125,000	68,200	101,000	111,000	68,900	89,776	100,000	100,000	106,000	118,000
MEDN0068	S-(5-Adenosy)-L-Homocysteine	**383.1137888**	C14H20N6O5S	493,000	489,000	596,000	928,000	426,000	584,000	355,000	322,000	397,000	342,000	359,000	354,000	101,000	122,000	110,000	113,000	109,612	103,000	302,000	348,000	317,000
MEDN0070	Sarcosine	**88.03987847**	C3H7NO2	77,800	88,600	93,000	100,000	91,300	97,700	71,700	100,000	54,000	61,100	64,000	79,100	62,300	57,200	60,900	66,400	62,903	67,700	64,600	81,500	84,000
MEDN0074	γ-L-Glutamate-Cysteine	**249.0545426**	C8H14N2O5S	198,000	299,000	313,000	268,000	271,000	289,000	15,500	11,100	19,600	15,000	11,300	20,200	32,200	19,900	24,800	15,200	22,545	20,600	88,700	96,300	92,300
MEDN0085	Benzoylformic Acid	**149.0238941**	C8H6O3	9	9	9	9	9	9	37,100	55,500	19,700	14,400	46,000	50,100	73,500	64,800	63,000	42,700	58,081	46,400	40,000	36,200	35,100
MEDN0090	2-Methoxybenzoic Acid	**151.0395441**	C8H8O3	9	9	9	9	9	9	9,860	7,750	14,300	11,500	7,210	8,520	9,780	13,000	15,700	13,300	13,188	14,200	6,220	6,850	4,890
MEDN0093	3-Hydroxyanthranilic Acid	**152.0347931**	C7H7NO3	9	9	9	9	6,040	9	51,800	27,400	76,000	65,700	35,900	54,000	60,600	61,400	71,000	76,200	68,472	73,100	36,800	45,000	46,800
MEDN0113	Glycochenodeoxycholic Acid	**448.3063235**	C26H43NO5	167,000	187,000	194,000	225,000	181,000	203,000	363,000	397,000	404,000	353,000	317,000	345,000	268,000	273,000	311,000	391,000	304,676	281,000	248,000	307,000	318,000
MEDN0120	Dulcitol	**181.0712382**	C6H14O6	196,000	187,000	200,000	190,000	206,000	195,000	143,000	118,000	162,000	124,000	159,000	153,000	147,000	181,000	179,000	131,000	168,840	205,000	108,000	124,000	134,000
MEDN0140	Xanthine	**151.0256254**	C5H4N4O2	61,500,000	62,100,000	77,000,000	65,700,000	68,000,000	78,600,000	73,900,000	65,100,000	82,600,000	69,400,000	74,100,000	78,500,000	75,000,000	69,300,000	63,900,000	80,400,000	71,433,200	68,500,000	63,700,000	68,200,000	84,900,000
MEDN0141	2′-Deoxyuridine	**227.0668215**	C9H12N2O5	20,600	25,900	24,800	32,000	17,800	30,100	18,300	19,600	14,100	16,400	21,100	20,500	12,000	18,600	19,600	18,900	18,762	24,800	19,300	20,100	16,400
MEDN0149	7-Methylxanthine	**165.0412755**	C6H6N4O2	9	9	9	9	7,350	9	17,700	24,600	8,290	11,000	26,600	17,800	75,100	40,400	31,000	36,200	45,542	45,000	22,300	27,400	23,300
MEDN0153	Adenosine 5′-Monophosphate	**346.0552844**	C10H14N5O7P	60,600	56,500	62,600	43,400	41,600	62,900	26,300	24,000	16,300	33,200	28,700	29,400	7,430	8,280	5,840	5,570	7,339	9,580	14,900	12,400	15,800

MEDN0159	Flavin Adenine Dinucleotide	**784.1493347**	C27H33N9O15P2	196,000	215,000	244,000	234,000	242,000	276,000	411,000	355,000	440,000	317,000	356,000	588,000	77,200	51,600	62,800	47,000	58,405	53,400	146,000	309,000	312,000
MEDN0165	Inosine 5′-Monophosphate	**347.0393**	C10H13N4O8P	129,000	139,000	133,000	81,400	251,000	142,000	3,260,000	2,890,000	3,140,000	3,890,000	3,310,000	3,070,000	60,300	54,700	36,900	102,000	61,828	55,500	961,000	1,370,000	1,270,000
MEDN0198	Citric Acid	**191.0192026**	C6H8O7	325,000	324,000	199,000	185,000	198,000	216,000	13,000,000	7,240,000	13,400,000	15,600,000	11,900,000	16,900,000	5,510,000	4,630,000	3,610,000	3,650,000	4,217,800	3,690,000	7,280,000	7,870,000	8,470,000
MEDN0202	A-Ketoglutaric Acid	**145.0137233**	C5H6O5	9	9	9	9	9	9	592,000	403,000	669,000	791,000	536,000	560,000	139,000	176,000	138,000	146,000	151,388	157,000	228,000	298,000	284,000
MEDN0203	Cis-Aconitic Acid	**173.0086379**	C6H6O6	5,430	4,520	5,420	6,020	6,620	7,310	12,800	12,600	13,600	9,290	13,400	15,200	17,500	19,900	12,000	18,100	16,412	14,500	10,200	24,400	15,000
MEDN0206	Citramalic Acid	**147.0293734**	C5H8O5	527,000	567,000	560,000	777,000	740,000	635,000	1,140,000	1,020,000	974,000	1,020,000	1,320,000	1,350,000	923,000	949,000	1,040,000	948,000	974,970	1,010,000	823,000	804,000	932,000
MEDN0207	6-Hydroxymelatonin	**247.1082924**	C13H16N2O3	9	9	9	9	9	9	4,000	3,060	3,250	2,580	5,470	5,650	3,700	3,700	4,310	4,750	4,321	5,150	2,700	2,420	3,930
MEDN0209	Tryptamine	**159.0922484**	C10H12N2	388,000	412,000	425,000	393,000	432,000	422,000	631,000	574,000	656,000	616,000	609,000	700,000	428,000	510,000	533,000	488,000	492,338	504,000	454,000	531,000	553,000
MEDN0210	1,5-Anhydro-D-Glucitol	**163.0606735**	C6H12O5	34,100	46,800	49,200	45,200	45,900	53,800	17,500	14,400	14,600	22,700	13,200	22,600	24,400	25,800	20,800	23,700	23,319	21,800	26,100	24,400	25,200
MEDN0211	D-Arabitol	**151.0606735**	C5H12O5	108,000	122,000	142,000	128,000	114,000	134,000	44,600	32,600	36,600	21,000	48,500	84,300	20,300	25,500	23,000	17,800	21,417	20,500	25,300	20,200	35,300
MEDN0213	D-Sorbitol	**181.0712382**	C6H14O6	154,000	144,000	121,000	156,000	144,000	174,000	90,700	76,600	104,000	74,000	110,000	88,300	76,900	108,000	108,000	75,000	95,474	109,000	87,400	95,200	71,500
MEDN0220	D-Glucose	**179.0555881**	C6H12O6	1,010,000	1,450,000	1,550,000	1,560,000	1,160,000	1,350,000	310,000	230,000	330,000	300,000	353,000	339,000	183,000	277,000	279,000	252,000	247,752	247,000	208,000	240,000	245,000
MEDN0222	D-Melezitose	**503.1612349**	C18H32O16	73,000	60,300	52,500	72,400	80,300	83,700	27,600	28,600	24,700	20,800	33,900	29,800	18,600	23,000	19,000	20,600	19,738	17,500	15,900	23,200	21,500

MEDN0224	D-Trehalose	**341.1084115**	C12H22O11	81,200	79,000	86,600	106,000	97,400	74,500	105,000	109,000	109,000	108,000	97,800	103,000	120,000	125,000	118,000	128,000	123,340	125,000	109,000	156,000	140,000
MEDN0227	D-Glucose 6-Phosphate	**259.0219186**	C6H13O9P	86,700,000	63,600,000	56,200,000	73,900,000	72,400,000	92,300,000	16,900,000	15,800,000	14,500,000	16,400,000	19,100,000	18,700,000	873,000	1,620,000	1,640,000	1,050,000	1,342,850	1,530,000	5,670,000	7,710,000	6,760,000
MEDN0230	Lactulose	**341.1084115**	C12H22O11	81,200	79,000	86,600	106,000	97,400	74,500	105,000	109,000	109,000	108,000	97,800	103,000	120,000	125,000	118,000	128,000	123,340	125,000	109,000	156,000	140,000
MEDN0231	L-Fucose	**163.0606735**	C6H12O5	34,100	46,800	49,200	45,200	45,900	53,800	17,500	14,400	14,600	22,700	13,200	22,600	24,400	25,800	20,800	23,700	23,319	21,800	26,100	24,400	25,200
MEDN0232	L-Rhamnose	**163.0606735**	C6H12O5	34,100	46,800	49,200	45,200	45,900	53,800	17,500	14,400	14,600	22,700	13,200	22,600	24,400	25,800	20,800	23,700	23,319	21,800	26,100	24,400	25,200
MEDN0234	Maltotriose	**503.1612349**	C18H32O16	73,000	60,300	52,500	72,400	80,300	83,700	27,600	28,600	24,700	20,800	33,900	29,800	18,600	23,000	19,000	20,600	19,738	17,500	15,900	23,200	21,500
MEDN0237	D-Glucoronic Acid	**193.0348527**	C6H10O7	349,000	401,000	535,000	475,000	400,000	416,000	118,000	95,800	123,000	129,000	129,000	112,000	66,900	95,300	78,300	64,200	77,471	82,600	81,500	96,600	90,900
MEDN0239	Gluconic Acid	**195.0505027**	C6H12O7	7,210	9,230	7,340	9,400	7,680	7,770	8,780	10,400	8,380	9,540	8,060	7,570	8,350	7,610	6,830	6,850	7,178	6,250	7,970	8,720	7,140
MEDN0240	L-Gulonolactone	**177.039938**	C6H10O6	19,600	23,700	16,100	27,900	23,200	16,900	90,900	53,200	88,000	61,400	101,000	151,000	50,500	51,100	47,400	62,700	51,444	45,600	45,800	77,200	47,300
MEDN0241	Vitamin D3	**383.331416**	C27H44O	38,100	31,800	24,000	25,800	22,000	21,900	16,900	11,700	16,400	19,400	14,000	22,800	13,500	9,510	14,100	8,400	12,479	17,000	39,300	20,400	20,700
MEDN0244	Orotic Acid	**155.0093066**	C5H4N2O4	14,800	23,100	10,900	12,700	12,700	21,600	73,100	24,300	78,100	108,000	62,400	92,700	51,300	39,300	32,200	30,900	38,756	40,200	49,700	51,900	61,400
MEDN0245	Pantothenate	**218.1028726**	C9H17NO5	24,200,000	30,900,000	31,000,000	30,900,000	28,700,000	29,000,000	26,500,000	25,300,000	26,400,000	21,300,000	28,300,000	31,100,000	29,900,000	32,700,000	33,100,000	31,200,000	32,147,200	33,900,000	22,200,000	30,100,000	31,400,000
MEDN0276	1-Naphthylacetic Acid	**185.0602796**	C12H10O2	9	9	9	9	9	9	60,200	166,000	9	9	68,800	65,800	180,000	59,500	793,000	1,010,000	429,453	108,000	142,000	145,000	144,000

MEDN0280	2-Aminoethanesulfonic Acid	**124.0068641**	C2H7NO3S	190,000,000	183,000,000	184,000,000	170,000,000	186,000,000	183,000,000	2,750,000	2,510,000	2,940,000	3,070,000	2,760,000	2,490,000	5,000,000	4,880,000	4,810,000	4,730,000	4,824,340	4,710,000	7,690,000	9,340,000	9,040,000
MEDN0283	2-Hydroxybutanoic Acid	**103.0395441**	C4H8O3	396,000	306,000	373,000	346,000	416,000	414,000	1,320,000	1,060,000	1,450,000	1,280,000	1,400,000	1,430,000	1,420,000	1,480,000	1,330,000	1,400,000	1,428,580	1,520,000	868,000	1,030,000	1,080,000
MEDN0284	2-Hydroxyisocaproic Acid	**131.0708442**	C6H12O3	28,700	20,600	21,400	61,400	32,800	32,100	192,000	87,900	153,000	133,000	161,000	425,000	86,700	101,000	88,000	131,000	103,329	110,000	75,400	98,000	93,400
MEDN0285	2-Methylsuccinic Acid	**131.0344587**	C5H8O4	1,050,000	1,140,000	1,070,000	1,190,000	1,260,000	1,180,000	2,610,000	2,290,000	2,750,000	2,470,000	2,750,000	2,800,000	4,070,000	4,510,000	4,510,000	4,940,000	4,580,820	4,870,000	2,070,000	2,550,000	2,680,000
MEDN0287	3,5-Dimethoxy-4-Hydroxycinnamic Acid	**223.0606735**	C11H12O5	9	122	9	9	9	9	36,400	28,400	34,000	27,800	33,700	57,900	95,200	158,000	148,000	151,000	136,748	131,000	37,800	58,400	54,400
MEDN0290	3-Hydroxy-3-Methyl Butyric Acid	**117.0551942**	C5H10O3	81,700	76,100	74,800	107,000	70,500	67,200	159,000	157,000	138,000	151,000	181,000	168,000	160,000	181,000	207,000	138,000	176,868	197,000	118,000	130,000	119,000
MEDN0292	3-Hydroxybutyrate	**103.0395441**	C4H8O3	9,220,000	10,600,000	10,100,000	9,390,000	9,570,000	10,100,000	3,680,000	3,560,000	3,790,000	3,510,000	3,710,000	3,810,000	5,200,000	5,110,000	5,560,000	5,290,000	5,327,340	5,480,000	4,540,000	5,490,000	5,680,000
MEDN0293	3-Hydroxypropanoic Acid	**89.02389405**	C3H6O3	35,200	43,100	39,300	39,400	33,500	64,600	25,100	32,800	23,800	22,000	23,900	23,000	21,500	23,200	23,600	15,600	20,960	20,900	20,600	23,600	24,900
MEDN0301	Caffeic Acid	**179.0344587**	C9H8O4	166,000	180,000	166,000	202,000	185,000	171,000	207,000	194,000	242,000	174,000	208,000	217,000	205,000	249,000	234,000	233,000	229,676	227,000	193,000	219,000	229,000
MEDN0314	Glutaric Acid	**131.0344587**	C5H8O4	1,050,000	1,140,000	1,070,000	1,190,000	1,260,000	1,180,000	2,610,000	2,290,000	2,750,000	2,470,000	2,750,000	2,800,000	4,070,000	4,510,000	4,510,000	4,940,000	4,580,820	4,870,000	2,070,000	2,550,000	2,680,000
MEDN0316	Guanidinoethyl Sulfonate	**166.0286622**	C3H9N3O3S	63,000	83,400	83,400	71,000	78,400	74,100	37,200	29,100	42,400	26,900	37,100	50,400	40,100	39,100	40,200	10,700	33,497	37,400	23,300	55,400	38,100
MEDN0320	Hydrocinnamic Acid	**149.0602796**	C9H10O2	13,400	14,300	13,600	15,500	19,400	9,770	18,000	14,100	19,500	22,600	15,200	18,700	19,200	16,600	15,300	11,100	15,760	16,700	15,400	17,000	16,600

MEDN0325	L-Lactic Acid	**89.02389405**	C3H6O3	2,490,000	2,430,000	2,630,000	2,540,000	2,600,000	2,590,000	3,180,000	3,000,000	3,310,000	3,300,000	3,300,000	2,970,000	3,080,000	2,930,000	2,760,000	2,810,000	2,886,480	2,860,000	2,380,000	2,940,000	3,000,000
MEDN0333	Malonicacid	**103.0031586**	C3H4O4	9,140,000	11,100,000	10,200,000	9,880,000	9,540,000	10,100,000	3,700,000	3,510,000	3,820,000	3,570,000	3,550,000	4,030,000	5,280,000	5,250,000	5,480,000	5,220,000	5,347,940	5,510,000	4,440,000	5,580,000	5,890,000
MEDN0334	Mandelic Acid	**151.0395441**	C8H8O3	78,200	29,500	25,900	89,600	68,600	99,900	543,000	322,000	830,000	628,000	401,000	535,000	632,000	683,000	714,000	693,000	686,160	710,000	390,000	438,000	443,000
MEDN0335	Methylmalonic Acid	**117.0188087**	C4H6O4	1,180,000	1,170,000	951,000	1,400,000	1,010,000	1,210,000	188,000,000	179,000,000	190,000,000	173,000,000	200,000,000	199,000,000	240,000,000	258,000,000	279,000,000	243,000,000	257,160,000	266,000,000	135,000,000	168,000,000	170,000,000
MEDN0338	Phenyllactate(Pla)	**165.0551942**	C9H10O3	58,800	32,300	29,900	75,900	96,700	63,300	1,940,000	1,030,000	2,620,000	1,310,000	1,960,000	2,760,000	1,340,000	1,630,000	1,490,000	1,710,000	1,555,380	1,600,000	888,000	1,080,000	1,100,000
MEDN0339	Phenylpyruvic Acid	**163.0395441**	C9H8O3	9	9	9	9	9	9	24,700,000	8,520,000	43,900,000	35,400,000	12,700,000	23,100,000	1,680,000	3,160,000	2,870,000	2,030,000	2,606,160	3,290,000	7,280,000	8,410,000	8,580,000
MEDN0340	Pyrrole-2-Carboxylic Acid	**110.0242284**	C5H5NO2	9	9	9	9	9	9	31,400	16,800	31,900	27,200	34,300	46,700	32,900	26,900	35,000	35,000	31,962	30,000	21,500	25,700	17,800
MEDN0342	Sebacate	**201.1127091**	C10H18O4	7,430	6,530	7,650	6,770	7,460	7,540	8,580	8,180	7,330	9,000	8,670	9,700	19,800	15,300	19,900	21,600	18,876	17,700	10,700	13,100	11,400
MEDN0343	Shikimic Acid	**173.0450234**	C7H10O5	61,500	62,100	74,400	87,700	82,400	70,100	188,000	147,000	268,000	158,000	183,000	184,000	351,000	442,000	343,000	496,000	421,102	473,000	202,000	226,000	247,000
MEDN0407	3-Aminosalicylic Acid	**152.0347931**	C7H7NO3	199,000	177,000	304,000	209,000	194,000	298,000	207,000	147,000	292,000	227,000	187,000	182,000	283,000	201,000	148,000	293,000	222,854	190,000	197,000	148,000	289,000
MEDN0411	Neopterin	**252.0733039**	C9H11N5O4	1,170,000	1,130,000	1,300,000	1,350,000	1,230,000	1,350,000	1,370,000	1,300,000	1,380,000	1,470,000	1,420,000	1,280,000	1,380,000	1,390,000	1,510,000	1,580,000	1,493,760	1,610,000	1,030,000	1,490,000	1,520,000
MEDN0412	Biopterin	**236.0783892**	C9H11N5O3	9	9	9	9	9	9	46,800	50,000	32,400	50,300	52,600	48,800	58,800	62,700	49,500	66,300	60,864	66,900	29,400	26,600	49,100

MEDN0413	Ethylmalonate	**131.0344587**	C5H8O4	1,050,000	1,140,000	1,070,000	1,190,000	1,260,000	1,180,000	2,610,000	2,290,000	2,750,000	2,470,000	2,750,000	2,800,000	4,070,000	4,510,000	4,510,000	4,940,000	4,580,820	4,870,000	2,070,000	2,550,000	2,680,000
MEDN0417	(Rs)-Mevalonic Acid	**147.0657589**	C6H12O4	622,000	703,000	707,000	865,000	675,000	738,000	51,600	46,100	65,400	59,500	52,700	34,200	42,400	85,200	34,300	74,400	61,191	69,600	160,000	175,000	179,000
MEDN0432	5-Hydroxyhexanoic Acid	**131.0708442**	C6H12O3	120,000	132,000	112,000	125,000	139,000	120,000	206,000	210,000	188,000	174,000	223,000	232,000	554,000	498,000	548,000	533,000	538,996	562,000	215,000	280,000	269,000
MEDN0439	N-Acetylthreonine	**160.0610078**	C6H11NO4	15,700	18,600	26,000	17,900	14,100	24,100	104,000	62,600	112,000	91,600	114,000	139,000	55,500	65,400	55,200	60,200	57,679	52,100	56,000	68,200	73,200
MEDN0444	Hypoxanthine-9-β-D-Arabinofuranoside	**267.0729695**	C10H12N4O5	219,000,000	194,000,000	185,000,000	184,000,000	226,000,000	210,000,000	581,000,000	614,000,000	573,000,000	569,000,000	509,000,000	641,000,000	498,000,000	528,000,000	588,000,000	559,000,000	539,994,000	527,000,000	503,000,000	565,000,000	549,000,000
MEDN0445	Orotidine	**287.0515654**	C10H12N2O8	27,900	32,200	40,400	27,600	22,800	24,200	108,000	100,000	105,000	102,000	119,000	113,000	109,000	104,000	104,000	95,200	109,326	135,000	72,400	70,400	107,000
MEDN0455	Guanine	**150.0416098**	C5H5N5O	9	9	1,190	1,220	9	9	34,500	30,600	36,600	36,100	32,400	36,500	33,000	21,600	24,500	29,400	27,410	28,600	25,000	30,000	28,700
MEDN0461	D-Sedoheptuiose 7-Phosphate	**287.0168332**	C7H13BaO10P	216,000	211,000	227,000	249,000	214,000	274,000	168,000	156,000	162,000	150,000	183,000	188,000	179,000	198,000	169,000	171,000	181,500	190,000	127,000	179,000	170,000
MEDN0463	D-Fructose 6-Phosphate-Disodium Salt	**259**	C6H11Na2O9P	9	9	9	9	9	9	4,810,000	3,950,000	4,320,000	5,160,000	5,200,000	5,420,000	293,000	428,000	442,000	317,000	395,448	497,000	1,690,000	2,110,000	2,160,000
MEDN0470	Oxoadipic Acid	**159.0293734**	C6H8O5	9	9	9	9	9	9	59,800	47,600	48,200	87,200	47,600	68,400	33,900	33,800	36,100	47,600	39,111	44,100	40,800	29,100	32,100
MEDN0478	Aminomalonic Acid	**118.0140576**	C3H5NO4	43,200	61,200	38,700	46,900	39,000	47,000	7,130,000	6,770,000	7,200,000	6,530,000	7,410,000	7,720,000	9,270,000	10,100,000	10,900,000	9,340,000	10,057,860	10,600,000	4,910,000	6,360,000	6,400,000
MEDN0487	Allysine	**144.0660932**	C6H11NO3	5,070	6,190	3,520	9,930	3,880	12,100	73,100	58,900	62,600	96,400	59,400	88,300	38,700	25,800	24,000	25,900	31,526	43,300	34,300	48,400	28,900

MEDN0496	N-Acetylmethionine	**190.0538143**	C7H13NO3S	65,500	59,000	61,800	83,000	70,000	78,200	537,000	361,000	661,000	545,000	520,000	600,000	173,000	190,000	171,000	153,000	178,384	205,000	209,000	239,000	245,000
MEDN0498	Ribulose-5-Phosphate	**229.0113539**	C5H11O8P	24,100,000	20,500,000	19,700,000	17,800,000	22,700,000	21,000,000	2,330,000	1,870,000	2,340,000	2,980,000	2,130,000	2,340,000	1,770,000	1,900,000	1,720,000	1,920,000	1,729,760	1,350,000	2,130,000	2,630,000	2,030,000
MEDN0499	Argininosuccinic acid	**289.1148343**	C10H18N4O6	9	9	9	9	9	9	280,000	235,000	236,000	286,000	301,000	342,000	123,000	172,000	132,000	175,000	160,848	202,000	206,000	239,000	245,000
MEDN0501	Acetyl Tryptophan	**245.0926423**	C13H14N2O3	28,100	38,200	45,900	43,100	38,200	43,600	69,200	65,400	62,700	66,300	77,600	73,800	147,000	149,000	133,000	165,000	147,358	142,000	79,300	109,000	94,600
MEDN0502	2-Deoxyribose 1-Phosphate	**213.0164393**	C5H11O7P	157,000	162,000	115,000	173,000	156,000	147,000	403,000	427,000	372,000	326,000	472,000	418,000	493,000	551,000	542,000	552,000	553,370	629,000	279,000	318,000	402,000
MEDN0506	N-Acetylglucosamine 1-Phosphate	**300.0484677**	C8H16NO9P	3,930,000	4,020,000	3,960,000	3,870,000	3,970,000	4,050,000	331,000	378,000	245,000	368,000	336,000	329,000	244,000	274,000	327,000	238,000	253,250	184,000	280,000	415,000	279,000
MEDN0530	Pimelic acid	**159.0657589**	C7H12O4	444,000	486,000	417,000	513,000	547,000	459,000	1,170,000	1,230,000	1,020,000	885,000	1,250,000	1,470,000	4,150,000	4,090,000	3,870,000	4,700,000	4,246,540	4,420,000	1,800,000	2,070,000	2,180,000
MEDN0531	D-Glucarate	**209.0297673**	C6H10O8	186,000	180,000	201,000	202,000	162,000	150,000	6,610	5,270	5,220	6,900	12,800	2,810	8,140	1,450	1,450	7,590	6,789	15,300	12,100	8,250	14,000
MEDN0533	Xanthosine	**283.0678841**	C10H12N4O6	66,700,000	76,100,000	75,700,000	74,200,000	76,800,000	74,500,000	59,300,000	56,500,000	61,200,000	47,900,000	60,600,000	70,600,000	57,300,000	64,000,000	61,900,000	61,600,000	61,845,800	64,500,000	55,600,000	68,400,000	69,100,000
MEDN0538	UDP-glucose	**565.0472206**	C15H24N2O17P2	31,900	28,900	21,000	29,000	23,200	16,100	26,900	25,800	18,000	29,000	28,400	33,500	9	9	9	9	9	9	12,800	11,800	15,800
MEDN0555	Hydroxyphenyllactic acid	**181.0501088**	C9H10O4	227,000	171,000	163,000	163,000	207,000	223,000	1,130,000	783,000	1,430,000	824,000	1,150,000	1,460,000	520,000	780,000	679,000	768,000	697,118	739,000	594,000	639,000	711,000
MEDN0561	N-Acetylaspartylglutamic acid	**303.0828655**	C11H16N2O8	5,840,000	10,200,000	10,600,000	9,390,000	7,230,000	8,790,000	1,140,000	799,000	1,300,000	1,350,000	1,190,000	1,070,000	470,000	472,000	400,000	451,000	474,238	578,000	1,030,000	1,400,000	1,630,000
MEDN0570	L-Erythrulose	**119.0344587**	C4H8O4	9	9	9	9	9	9	61,500	57,900	56,200	58,900	75,000	59,600	118,000	124,000	120,000	114,000	121,166	129,000	64,800	80,700	82,000

MEDN0579	N-lactoyl-phenylalanine	**236.092308**	C12H15NO4	2,130,000	2,810,000	2,940,000	2,940,000	2,580,000	2,710,000	5,970,000	5,720,000	5,810,000	4,700,000	6,200,000	7,420,000	13,800,000	14,600,000	13,900,000	14,400,000	14,435,800	15,400,000	6,350,000	7,980,000	8,330,000
MEDN0587	N-Acetylvaline	**158.0817433**	C7H13NO3	172,000	272,000	311,000	302,000	222,000	265,000	173,000	141,000	210,000	143,000	178,000	193,000	236,000	269,000	268,000	242,000	254,714	258,000	196,000	227,000	198,000
MEDN0593	D-Malic acid	**133.0137233**	C4H6O5	43,900,000	46,500,000	43,100,000	43,100,000	41,400,000	43,600,000	583,000	443,000	585,000	543,000	649,000	696,000	1,660,000	1,520,000	1,670,000	1,460,000	1,604,120	1,700,000	6,130,000	7,770,000	7,570,000
MEDN0602	deoxyguanosine 5′-mono-phosphate(dGMP)	**346.0552844**	C10H14N5O7P	60,600	56,500	62,600	43,400	41,600	62,900	26,300	24,000	16,300	33,200	28,700	29,400	7,430	8,280	5,840	5,570	7,339	9,580	14,900	12,400	15,800
MEDN0606	5-Methoxytryptophan	**190.0868287**	C11H13NO2	113,000	109,000	94,000	88,800	86,600	95,400	98,300	82,800	110,000	91,400	96,500	111,000	51,600	57,800	58,700	55,500	55,987	56,200	81,600	83,900	92,300
MEDN0616	O-Acetyl-L-serine	**146.0453578**	C5H9NO4	909,000	974,000	1,020,000	1,070,000	1,080,000	1,030,000	1,600,000	1,360,000	1,680,000	1,700,000	1,650,000	1,600,000	1,640,000	1,860,000	1,770,000	1,730,000	1,771,520	1,860,000	1,310,000	1,600,000	1,650,000
MEDN0622	Porphobilinogen	**225.0875569**	C10H14N2O4	26,600	30,600	35,000	20,700	20,000	41,300	34,600	23,000	53,200	18,700	35,500	42,700	33,300	28,100	63,600	91,700	52,414	45,400	69,800	89,000	61,300
MEDN0651	Succinic anhydride	**99.00824399**	C4H4O3	873,000	1,190,000	1,120,000	1,210,000	1,100,000	1,020,000	1,570,000	2,040,000	1,340,000	1,830,000	1,360,000	1,290,000	2,780,000	2,090,000	2,070,000	2,250,000	2,192,260	1,780,000	1,760,000	2,220,000	1,960,000
MEDN0655	Hydantoin-5-propionic acid	**171.0406067**	C6H8N2O4	7,940	9,380	12,100	11,700	5,080	7,810	4,990	2,810	5,680	3,600	7,790	5,080	7,670	6,600	4,350	8,620	5,907	2,300	5,140	6,920	5,140
MEDN0657	D-Xylulose 5-phosphate	**229.0113539**	C5H11O8P	24,100,000	20,500,000	19,700,000	17,800,000	22,700,000	21,000,000	2,330,000	1,870,000	2,340,000	2,980,000	2,130,000	2,340,000	1,770,000	1,900,000	1,720,000	1,920,000	1,729,760	1,350,000	2,130,000	2,630,000	2,030,000
MEDN0661	2-hydroxy-2-(4-hydroxy-3-methoxyphenyl)acetic acid	**197.0450234**	C9H10O5	14,100	18,700	14,100	15,500	17,400	17,800	22,500	21,800	24,300	20,500	22,700	23,400	19,800	20,400	20,500	23,200	21,469	23,400	15,900	15,200	21,400
MEDN0662	Gamma-Glu-Leu	**259.1294217**	C11H20N2O5	66,300	76,000	88,700	84,000	85,000	90,500	612,000	523,000	685,000	544,000	624,000	684,000	718,000	866,000	842,000	778,000	810,266	847,000	418,000	526,000	494,000
MEDN0682	Octadecanamide	**282.2797148**	C18H37NO	21,100	24,300	24,300	27,900	15,600	25,000	23,200	17,700	26,700	19,100	24,500	27,800	26,300	24,000	14,600	22,200	21,086	18,300	10,600	14,100	14,400

MEDN0720	N-(2-Methylbenzoyl)glycine	**192.0660932**	C10H11NO3	52,600	60,000	61,600	59,200	56,000	59,300	55,900	49,600	60,700	55,100	56,400	57,800	64,200	67,900	64,300	70,000	66,216	64,600	54,700	57,200	64,700
MEDN0725	2,4-Hexadienoic acid	**111.0446295**	C6H8O2	3,310,000	3,110,000	3,390,000	2,940,000	2,870,000	3,360,000	645,000	619,000	654,000	751,000	614,000	589,000	669,000	587,000	591,000	653,000	610,634	554,000	624,000	804,000	685,000
MEDN0741	Methanesulfonic acid	**94.98031499**	CH4O3S	319,000	346,000	272,000	292,000	327,000	320,000	26,400	20,300	23,900	44,300	21,900	21,500	30,300	45,700	53,500	57,900	46,491	45,000	62,800	56,600	44,600
MEDN0793	Prostaglandin E2	**351.2171741**	C20H32O5	31,600	27,700	21,300	36,600	39,200	31,300	638,000	1,240,000	9	9	1,360,000	588,000	792,000	1,120,000	626,000	2,840,000	1,107,370	154,000	439,000	521,000	530,000
MEDN0818	N-acetyl-beta-alanine	**130.0504432**	C5H9NO3	859,000	895,000	989,000	989,000	888,000	940,000	1,700,000	1,470,000	1,870,000	1,730,000	1,640,000	1,810,000	679,000	660,000	694,000	771,000	700,544	699,000	845,000	1,030,000	962,000
MEDN0820	3-Hydroxyglutaric acid	**147.0293734**	C5H8O5	248,000	181,000	225,000	271,000	221,000	250,000	403,000	329,000	414,000	420,000	450,000	400,000	367,000	321,000	335,000	316,000	332,802	325,000	278,000	302,000	364,000
MEDN0824	7-ketodeoxycholic acid	**405.2641243**	C24H38O5	6,710	6,160	6,180	8,110	7,060	8,270	18,300	17,100	21,400	15,500	17,600	20,000	14,400	19,600	18,300	22,100	18,260	16,900	15,100	15,800	15,300
MEDN0827	N-acetylornithine	**173.0926423**	C7H14N2O3	5,710,000	6,530,000	6,510,000	6,650,000	5,950,000	6,240,000	2,850,000	2,700,000	3,010,000	2,760,000	2,860,000	2,910,000	3,400,000	3,580,000	3,340,000	3,230,000	3,387,040	3,380,000	2,800,000	3,130,000	3,110,000
MEDN0832	D-Glucosamine 6-Phosphate	**258.037903**	C6H14NO8P	9,030,000	9,360,000	9,510,000	9,940,000	9,790,000	9,920,000	1,090,000	864,000	1,040,000	1,370,000	1,110,000	1,070,000	750,000	799,000	831,000	769,000	778,792	745,000	933,000	1,180,000	1,160,000
MEDN0833	3-Dehydroshikimate	**171.0293734**	C7H8O5	79,800	111,000	123,000	98,900	99,100	112,000	98,800	96,600	98,500	96,100	111,000	91,700	89,100	88,400	87,200	93,700	88,841	85,800	90,900	87,700	94,400
MEDN0841	3-Ureidopropionate	**131.0456921**	C4H8N2O3	393,000	428,000	429,000	429,000	458,000	417,000	531,000	736,000	382,000	444,000	488,000	604,000	375,000	400,000	367,000	430,000	396,734	412,000	474,000	584,000	613,000
MEDN0854	Mono-Methyl Glutarate	**145.0501088**	C6H10O4	89,200	96,700	121,000	116,000	128,000	104,000	81,500	71,400	95,100	74,900	70,700	95,400	91,600	95,200	102,000	92,600	90,123	69,000	3,660	3,370	4,630
MEDN1004	L-Sepiapterin	**236.0783892**	C9H11N5O3	77,000	94,600	85,300	71,700	156,000	80,100	7,080,000	6,520,000	7,340,000	6,990,000	6,210,000	8,350,000	8,310,000	10,100,000	9,380,000	9,500,000	9,415,960	9,800,000	4,480,000	6,180,000	6,370,000

MEDN1006	Uric acid	**167.02054**	C5H4N4O3	1,560,000	1,510,000	1,580,000	1,530,000	1,730,000	1,660,000	2,520,000	1,700,000	3,430,000	2,520,000	2,000,000	2,940,000	1,570,000	2,350,000	2,250,000	1,750,000	2,046,620	2,310,000	1,990,000	2,100,000	2,360,000
MEDN1011	D-Mannitol	**181.0712382**	C6H14O6	196,000	187,000	200,000	190,000	206,000	195,000	143,000	118,000	162,000	124,000	159,000	153,000	147,000	181,000	179,000	131,000	168,840	205,000	108,000	124,000	134,000
MEDN1024	D-piperidine acid	**128.0711786**	C6H11NO2	350,000	450,000	423,000	374,000	362,000	403,000	1,140,000	1,010,000	1,060,000	1,150,000	1,290,000	1,210,000	3,410,000	3,550,000	3,700,000	3,110,000	3,491,440	3,680,000	1,460,000	1,740,000	1,830,000
MEDN1029	Glycyl-L-phenylalanine	**221.0926423**	C11H14N2O3	42,500	31,400	40,300	45,300	49,700	50,100	728,000	614,000	928,000	732,000	680,000	685,000	688,000	815,000	843,000	799,000	794,816	830,000	438,000	542,000	624,000
MEDN1039	Indole-4-carboxaldehyde	**144.0449638**	C9H7NO	6,530	7,140	9,370	8,770	6,920	8,390	817,000	644,000	677,000	834,000	1,100,000	833,000	632,000	211,000	205,000	255,000	326,814	332,000	325,000	379,000	390,000
MEDN1052	(S) -2-Hydroxyglutaric acid	**147.0293734**	C5H8O5	527,000	567,000	560,000	777,000	740,000	635,000	1,140,000	1,020,000	974,000	1,020,000	1,320,000	1,350,000	923,000	949,000	1,040,000	948,000	974,970	1,010,000	823,000	804,000	932,000
MEDN1055	D-Calcium Pantothenate	**437.2135453**	C18H34N2O10	163,000	252,000	236,000	243,000	197,000	210,000	221,000	185,000	218,000	145,000	260,000	296,000	271,000	331,000	269,000	250,000	288,298	320,000	195,000	249,000	272,000
MEDN1056	Iminodiacetic acid	**132.0297077**	C4H7NO4	6,930,000	9,180,000	10,100,000	8,200,000	7,870,000	8,520,000	11,100	15,800	9,410	10,500	11,200	8,540	37,200	56,400	56,300	62,200	53,932	57,400	44,100	59,900	46,200
MEDN1060	L-Isoisoleucine	**130.0868287**	C6H13NO2	31,000,000	33,500,000	34,900,000	31,900,000	34,100,000	35,700,000	105,000,000	96,200,000	111,000,000	108,000,000	99,400,000	110,000,000	93,400,000	92,100,000	87,700,000	98,600,000	92,951,800	92,900,000	68,800,000	86,300,000	91,300,000
MEDN1061	Glycine deoxycholic acid	**448.3063235**	C26H43NO5	6,850	7,720	7,360	7,400	7,420	8,680	14,600	14,200	15,500	14,100	12,400	16,800	11,400	12,700	12,900	16,900	13,028	11,100	10,700	11,700	11,000
MEDN1064	N-arachidene glycine	**360.253894**	C22H35NO3	9	9	9	9	9	9	4,270	3,560	5,780	4,250	3,030	4,710	12,600	12,700	10,400	9,530	11,977	14,700	4,870	4,880	6,540
MEDN1085	Glutathione	**611.1441625**	C20H32N6O12S2	6,480,000	8,850,000	8,930,000	6,710,000	6,490,000	7,290,000	213,000	344,000	209,000	120,000	209,000	183,000	11,200	23,000	19,000	11,800	17,240	21,300	312,000	366,000	379,000

MEDN1087	UDP-D-galactose	**565.04845**	C15H24N2O17P2	46,300	36,100	19,200	23,600	53,000	16,500	28,300	35,800	23,800	18,600	32,700	30,400	9	9	9	9	9	9	12,900	15,600	14,300
MEDN1098	2-ethyl-2-hydroxybutyric acid	**131.07071**	C6H12O3	111,000	110,000	97,300	105,000	121,000	107,000	197,000	195,000	172,000	177,000	192,000	248,000	525,000	489,000	507,000	538,000	519,122	536,000	196,000	273,000	272,000
MEDN1108	1,6-anhydro-β-D-glucose	**161.0450234**	C6H10O5	92,800	98,400	124,000	120,000	85,900	96,100	132,000	136,000	137,000	142,000	112,000	132,000	123,000	99,100	99,600	102,000	108,244	118,000	96,800	92,200	109,000
MEDN1110	1,11-undecylic acid	**243.2**	C13H24O4	1,970,000	1,920,000	1,980,000	1,980,000	1,910,000	1,970,000	2,010,000	2,000,000	2,030,000	2,030,000	1,910,000	2,080,000	2,080,000	2,080,000	2,140,000	2,220,000	2,123,960	2,110,000	1,590,000	2,000,000	2,040,000
MEDN1126	Gamma-Mercholic Acid	**407.2797744**	C24H40O5	4,370,000	4,470,000	4,440,000	4,730,000	4,390,000	4,710,000	10,100,000	9,770,000	11,700,000	9,410,000	8,670,000	10,800,000	7,430,000	8,870,000	9,020,000	10,400,000	8,930,560	8,880,000	8,010,000	8,520,000	8,460,000
MEDN1129	Erythrose	**119.0344587**	C4H8O4	9	9	9	9	9	9	61,500	57,900	56,200	58,900	75,000	59,600	118,000	124,000	120,000	114,000	121,166	129,000	64,800	80,700	82,000
MEDN1135	Heparin	**315.2899451**	C19H40O3	28,200	34,000	27,700	19,800	27,800	33,500	108,000	158,000	26,500	39,400	173,000	144,000	75,000	58,000	69,600	155,000	79,550	39,800	52,200	71,400	70,800
MEDN1145	P-chlorophenylalanine	**198.0322487**	C9H10ClNO2	3,370,000	3,500,000	3,520,000	3,590,000	3,730,000	3,710,000	2,710,000	2,690,000	2,600,000	2,860,000	2,640,000	2,750,000	2,730,000	2,650,000	2,640,000	2,660,000	2,663,200	2,630,000	2,520,000	2,920,000	3,000,000
MEDN1221	D-Mannose 6-phosphate	**259.0219186**	C6H13O9P	76,700,000	49,900,000	42,700,000	58,800,000	60,500,000	71,200,000	7,430,000	6,990,000	6,700,000	6,760,000	8,270,000	8,450,000	431,000	674,000	723,000	578,000	611,686	653,000	2,370,000	2,870,000	3,240,000
MEDN1226	2-amino-4-oxovaleric acid	**130.0504432**	C5H9NO3	859,000	895,000	989,000	989,000	888,000	940,000	1,700,000	1,470,000	1,870,000	1,730,000	1,640,000	1,810,000	679,000	660,000	694,000	771,000	700,544	699,000	845,000	1,030,000	962,000
MEDN1235	L-2-amino-6-oximelic acid	**188.0567506**	C7H11NO5	53,600	69,800	94,100	94,700	67,500	79,000	322,000	226,000	344,000	367,000	332,000	342,000	362,000	346,000	319,000	348,000	346,846	359,000	207,000	250,000	277,000
MEDN1246	3-(pyrazol-1-yl) -L-alanine	**154.0620148**	C6H9N3O2	8,250,000	7,160,000	6,730,000	7,620,000	7,980,000	8,350,000	10,300,000	10,000,000	10,100,000	9,850,000	10,600,000	11,100,000	5,960,000	7,840,000	7,710,000	7,150,000	7,366,620	8,180,000	6,100,000	7,270,000	7,480,000
MEDN1303	Mannose 1-phosphate	**259**	C6H13O9P	8,400,000	4,820,000	4,760,000	6,260,000	6,350,000	7,800,000	836,000	949,000	696,000	702,000	874,000	960,000	43,100	73,500	79,200	65,700	68,488	81,000	222,000	345,000	306,000

MEDN1306	N-Carbamoyl-L-aspartate	**175.04**	C5H8N2O5	94,900	119,000	110,000	111,000	124,000	117,000	56,800	45,500	64,900	64,400	52,100	57,200	72,100	73,500	70,500	77,100	72,096	67,300	52,900	49,200	64,100
MEDN1311	2-Hydroxycaprylic acid	**159**	C8H16O3	17,200	12,300	9,770	15,200	12,800	9,980	243,000	639,000	42,600	49,000	256,000	229,000	1,700,000	809,000	2,600,000	2,580,000	1,822,480	1,430,000	499,000	599,000	606,000
MEDN1317	3,3-Dimethylglutaric acid	**159**	C7H12O4	9	9	9	9	9	9	14,200	20,000	9	9	23,600	27,400	104,000	94,600	96,300	109,000	100,743	99,600	33,800	42,400	40,500
MEDN1326	2-Methylglutaric Acid	**145**	C6H10O4	9	9	9	9	9	9	20,300	21,100	17,300	19,600	17,000	26,400	60,500	63,700	65,800	60,300	62,969	64,500	29,800	35,600	41,800
MEDN1328	(S)-Leucic acid	**131**	C6H12O3	24,000	18,500	22,200	56,500	33,300	32,900	148,000	78,300	156,000	118,000	146,000	241,000	89,000	100,000	80,100	99,600	93,198	97,300	68,500	85,800	79,800
MEDP0002	D-Homocysteine	**136**	C4H9NO2S	366,000	269,000	277,000	297,000	352,000	320,000	246,000	222,000	263,000	266,000	218,000	258,000	150,000	116,000	132,000	114,000	130,610	141,000	196,000	164,000	181,000
MEDP0007	L-Cystine	**241**	C6H12N2O4S2	345,000	378,000	237,000	273,000	311,000	327,000	141,000	128,000	141,000	123,000	164,000	147,000	83,600	93,900	72,000	58,200	77,446	79,600	167,000	92,900	103,000
MEDP0009	L-Tyrosine	**182.1**	C9H11NO3	57,400,000	56,900,000	59,100,000	56,800,000	57,800,000	56,700,000	107,000,000	105,000,000	108,000,000	113,000,000	105,000,000	103,000,000	84,200,000	94,000,000	90,900,000	90,600,000	89,055,000	85,600,000	89,900,000	91,000,000	95,300,000
MEDP0010	L-Arginine	**175**	C6H14N4O2	1,510,000	2,160,000	1,750,000	2,550,000	1,840,000	2,580,000	4,350,000	3,740,000	4,680,000	4,180,000	4,190,000	4,960,000	4,000,000	4,340,000	4,180,000	4,250,000	4,220,160	4,320,000	3,760,000	3,960,000	4,090,000
MEDP0011	L-Lysine	**147**	C6H14N2O2	55,800,000	67,700,000	68,900,000	63,000,000	59,500,000	59,000,000	45,000,000	40,700,000	50,600,000	47,100,000	44,200,000	42,400,000	61,000,000	66,100,000	65,200,000	64,700,000	64,571,200	65,900,000	50,300,000	49,100,000	47,100,000
MEDP0012	L-Ornithine	**133**	C5H13ClN2O2	11,600,000	16,300,000	16,600,000	15,200,000	12,700,000	13,900,000	635,000	561,000	588,000	612,000	701,000	712,000	795,000	900,000	897,000	793,000	847,766	854,000	1,530,000	1,560,000	1,610,000
MEDP0013	L-Alanine	**90.048**	C3H7NO2	37,500,000	37,800,000	38,700,000	37,100,000	36,300,000	38,400,000	8,070,000	10,200,000	6,810,000	6,290,000	7,890,000	9,140,000	4,920,000	6,300,000	5,800,000	5,830,000	5,797,060	6,150,000	8,020,000	7,910,000	7,900,000

MEDP0014	L-Aspartic Acid	**134.1**	C4H7NO4	1,830,000	2,260,000	2,330,000	2,200,000	1,800,000	2,070,000	193,000	177,000	186,000	221,000	217,000	164,000	220,000	166,000	203,000	177,000	197,672	223,000	281,000	258,000	301,000
MEDP0015	L-Citrulline	**176.1**	C6H13N3O3	538,000	597,000	835,000	793,000	596,000	722,000	159,000	133,000	175,000	145,000	176,000	166,000	136,000	126,000	88,100	69,700	102,380	92,200	170,000	180,000	178,000
MEDP0016	L-Glutamic Acid	**148.1**	C5H9NO4	33,800,000	37,200,000	40,300,000	40,100,000	34,500,000	37,300,000	7,220,000	6,610,000	6,920,000	8,850,000	7,160,000	6,550,000	13,200,000	11,800,000	10,900,000	10,600,000	11,625,200	11,600,000	13,300,000	12,100,000	11,500,000
MEDP0017	L-Histidine	**156.1**	C6H9N3O2	103,000,000	98,100,000	88,800,000	93,900,000	105,000,000	97,500,000	54,000,000	45,900,000	60,700,000	62,100,000	51,400,000	50,100,000	39,600,000	37,400,000	36,800,000	37,200,000	37,270,200	35,300,000	42,800,000	45,300,000	47,000,000
MEDP0020	L-Methionine	**150**	C5H11NO2S	8,650,000	9,220,000	9,160,000	8,780,000	9,040,000	8,700,000	32,100,000	31,500,000	33,800,000	32,400,000	31,200,000	31,400,000	23,100,000	26,000,000	24,700,000	23,200,000	24,607,600	26,100,000	22,300,000	23,600,000	23,900,000
MEDP0021	L-Phenylalanine	**166**	C9H11NO2	349,000,000	340,000,000	348,000,000	349,000,000	347,000,000	350,000,000	349,000,000	348,000,000	348,000,000	349,000,000	349,000,000	353,000,000	357,000,000	353,000,000	355,000,000	351,000,000	354,272,000	356,000,000	349,000,000	355,000,000	352,000,000
MEDP0022	L-Proline	**116.1**	C5H9NO2	47,000,000	47,000,000	47,100,000	48,800,000	44,800,000	43,000,000	34,800,000	30,800,000	37,400,000	36,600,000	36,100,000	33,200,000	57,400,000	50,700,000	54,000,000	49,200,000	53,516,200	56,300,000	53,500,000	47,500,000	45,900,000
MEDP0025	L-Tryptophan	**205.1**	C11H12N2O2	16,900,000	17,600,000	17,000,000	16,300,000	16,900,000	17,900,000	56,700,000	51,500,000	62,500,000	49,700,000	57,000,000	62,900,000	28,200,000	46,100,000	43,500,000	39,900,000	40,423,000	44,400,000	37,000,000	38,900,000	41,300,000
MEDP0026	L-Valine	**118.1**	C5H11NO2	75,900,000	78,300,000	87,200,000	78,300,000	74,100,000	81,400,000	127,000,000	123,000,000	134,000,000	121,000,000	126,000,000	130,000,000	103,000,000	117,000,000	120,000,000	111,000,000	114,276,000	120,000,000	99,700,000	100,000,000	98,900,000
MEDP0034	3-N-Methyl-L-Histidine	**170**	C7H13N3O3	3,440,000	3,110,000	2,880,000	2,870,000	3,240,000	2,940,000	996,000	673,000	1,080,000	1,270,000	987,000	968,000	676,000	570,000	623,000	637,000	637,594	682,000	914,000	821,000	949,000
MEDP0035	5-Hydroxy-L-Tryptophan	**221.1**	C11H12N2O3	9	9	9	9	9	9	62,300	100,000	24,700	23,600	107,000	55,800	116,000	76,600	79,200	128,000	98,664	93,700	69,900	53,100	56,000

MEDP0036	5-Oxoproline	**130**	C5H7NO3	10,400,000	12,100,000	11,500,000	10,300,000	10,000,000	10,300,000	16,600,000	16,900,000	15,600,000	14,500,000	18,300,000	17,600,000	38,800,000	42,300,000	45,200,000	38,500,000	41,843,400	44,400,000	25,100,000	26,900,000	26,500,000
MEDP0039	Betaine	**118.1**	C5H11NO2	69,800,000	71,500,000	68,700,000	67,200,000	64,700,000	71,000,000	31,500,000	32,600,000	34,400,000	39,900,000	25,300,000	25,200,000	29,900,000	22,700,000	19,800,000	21,900,000	22,942,400	20,500,000	37,800,000	37,500,000	35,700,000
MEDP0041	D-Alanyl-D-Alanine	**161.2**	C6H12N2O3	86,700	44,000	44,000	63,800	59,600	52,800	102,000	77,000	122,000	139,000	82,600	89,600	96,400	99,900	71,400	86,900	83,723	64,000	73,300	86,200	79,700
MEDP0043	Glutathione Oxidized	**613.152**	C20H32N6O12S2	4,960,000	7,260,000	6,990,000	5,970,000	4,860,000	4,940,000	664,000	1,170,000	635,000	331,000	603,000	577,000	54,800	62,900	111,000	123,000	86,831	82,000	907,000	1,100,000	1,150,000
MEDP0044	Glutathione Reducedform	**308**	C10H17N3O6S	87,900,000	105,000,000	95,300,000	101,000,000	90,000,000	97,800,000	4,370,000	3,090,000	7,110,000	5,760,000	2,250,000	3,650,000	956,000	1,270,000	1,150,000	574,000	1,124,728	1,680,000	18,000,000	19,300,000	18,600,000
MEDP0047	Histamine	**112.08**	C5H9N3	14,900,000	13,500,000	13,500,000	16,500,000	16,200,000	15,300,000	8,500,000	7,410,000	10,200,000	9,140,000	7,960,000	7,820,000	6,760,000	6,710,000	6,380,000	6,300,000	6,579,480	6,750,000	7,440,000	7,980,000	8,560,000
MEDP0049	L-Asparagine Anhydrous	**133.054**	C4H8N2O3	1,170,000	1,230,000	1,120,000	1,330,000	1,290,000	1,160,000	1,250,000	1,290,000	1,280,000	1,240,000	1,280,000	1,160,000	1,270,000	1,340,000	1,270,000	1,310,000	1,273,180	1,180,000	1,370,000	1,310,000	1,270,000
MEDP0050	L-Carnosine	**227.2**	C9H14N4O3	244,000,000	210,000,000	199,000,000	204,000,000	234,000,000	240,000,000	125,000,000	104,000,000	132,000,000	133,000,000	125,000,000	132,000,000	82,900,000	77,800,000	73,400,000	79,600,000	78,487,600	78,800,000	104,000,000	121,000,000	120,000,000
MEDP0051	L-Cystathionine	**223**	C7H14N2O4S	9	21,100	36,300	42,800	6,580	26,500	18,500	19,300	28,800	21,800	9,570	13,000	30,200	27,600	24,800	22,300	25,079	20,500	19,800	17,200	23,300
MEDP0053	L-Dopa	**198.069**	C9H11NO4	9	9	9	9	9	9	120,000	120,000	81,800	76,700	190,000	129,000	309,000	188,000	197,000	176,000	209,362	177,000	108,000	125,000	119,000
MEDP0054	L-Glutamine	**147.069**	C5H10N2O3	154,000,000	158,000,000	166,000,000	163,000,000	159,000,000	154,000,000	3,880,000	2,840,000	4,340,000	6,140,000	3,060,000	3,050,000	9	9	9	9	9	9	12,700,000	12,900,000	15,200,000

MEDP0055	L-Homocitrulline	**190.1**	C7H15N3O3	140,000	126,000	165,000	164,000	155,000	155,000	66,500	50,400	83,000	78,200	58,500	62,300	86,400	79,800	78,500	74,000	74,961	56,200	79,000	84,800	87,800
MEDP0056	L-Homocystine	**269**	C8H16N2O4S2	393,000	368,000	370,000	441,000	365,000	394,000	2,640,000	2,660,000	2,510,000	2,460,000	2,780,000	2,790,000	1,720,000	1,900,000	1,980,000	1,900,000	1,898,720	1,990,000	1,800,000	1,870,000	1,930,000
MEDP0058	L-Saccharopine	**277**	C11H20N2O6	649,000	530,000	476,000	518,000	602,000	662,000	751,000	615,000	711,000	992,000	742,000	697,000	925,000	786,000	566,000	853,000	745,430	596,000	732,000	860,000	809,000
MEDP0060	Methionine Sulfoxide	**166**	C5H11NO3S	2,820,000	2,650,000	2,410,000	2,340,000	2,340,000	2,360,000	2,590,000	2,800,000	2,410,000	2,270,000	2,590,000	2,900,000	1,900,000	1,960,000	1,890,000	2,030,000	1,927,200	1,860,000	2,590,000	2,250,000	2,170,000
MEDP0061	N,N-Dimethylglycine	**104**	C4H9NO2	1,370,000	1,630,000	1,520,000	1,660,000	1,550,000	1,500,000	5,320,000	5,830,000	4,990,000	6,040,000	4,940,000	4,810,000	7,820,000	7,880,000	7,900,000	8,920,000	8,247,660	8,720,000	7,390,000	7,430,000	7,350,000
MEDP0062	N6-Acetyl-L-Lysine	**189.1160924**	C8H16N2O3	26,500,000	37,500,000	38,600,000	36,300,000	28,100,000	33,900,000	4,930,000	3,460,000	4,250,000	2,600,000	6,710,000	7,640,000	1,650,000	3,120,000	2,680,000	1,790,000	2,379,140	2,650,000	4,010,000	3,970,000	3,980,000
MEDP0063	N-Acetylaspartate	**176**	C6H9NO5	9	9	9	9	9	9	4,360,000	4,860,000	2,550,000	2,170,000	6,230,000	6,010,000	2,270,000	4,250,000	5,390,000	3,730,000	4,162,180	5,170,000	2,650,000	2,420,000	2,720,000
MEDP0065	N-Acetyl-L-Glutamic Acid	**190.1**	C7H11NO5	302,000	423,000	395,000	264,000	293,000	373,000	341,000	326,000	328,000	321,000	396,000	332,000	539,000	492,000	414,000	403,000	468,016	492,000	445,000	381,000	384,000
MEDP0066	N-Acetyl-L-Tyrosine	**224.1**	C11H13NO4	8,180	11,400	13,400	9	9	9	81,900	55,300	73,100	59,400	86,900	135,000	84,500	82,800	94,600	82,700	86,900	89,900	57,800	60,800	65,700
MEDP0067	N-Acetylmannosamine	**222**	C8H15NO6	502,000	627,000	403,000	429,000	529,000	488,000	600,000	615,000	565,000	463,000	710,000	647,000	458,000	524,000	546,000	460,000	484,800	436,000	512,000	605,000	513,000
MEDP0068	N-Acetylneuraminic Acid	**310.1**	C11H19NO9	33,400	48,500	29,800	34,600	40,600	37,700	33,600	36,000	49,100	26,000	20,400	36,600	21,700	24,900	18,600	23,800	23,196	27,000	51,300	26,100	46,400
MEDP0071	N-Glycyl-L-Leucine	**189.1**	C8H16N2O3	280,000	366,000	304,000	235,000	299,000	271,000	3,970,000	3,500,000	4,500,000	3,830,000	4,130,000	3,880,000	4,010,000	5,310,000	4,560,000	4,760,000	4,639,860	4,550,000	3,380,000	3,110,000	3,190,000
MEDP0072	N-Isovaleroylglycine	**160.1**	C7H13NO3	83,700	85,500	95,400	85,300	90,400	81,900	272,000	120,000	277,000	221,000	319,000	424,000	118,000	121,000	105,000	103,000	114,448	124,000	168,000	172,000	154,000

MEDP0075	Nα-Acetyl-L-Arginine	**217.1**	C8H16N4O3	236,000	178,000	221,000	242,000	198,000	216,000	291,000	158,000	237,000	182,000	373,000	504,000	250,000	206,000	228,000	202,000	225,398	241,000	212,000	191,000	168,000
MEDP0077	Phenylacetyl-L-Glutamine	**265.1**	C13H16N2O4	171,000	168,000	140,000	198,000	178,000	182,000	401,000	362,000	445,000	319,000	415,000	463,000	283,000	314,000	309,000	369,000	323,590	342,000	287,000	343,000	339,000
MEDP0078	Phe-Phe	**313.4**	C18H20N2O3	9	9	9	9	9	9	483,000	432,000	484,000	419,000	471,000	608,000	451,000	545,000	435,000	394,000	469,658	524,000	284,000	377,000	404,000
MEDP0080	S-Adenosyl-L-Methionine	**401.2**	C15H24N6O5S	15,500	23,700	22,300	30,600	16,100	28,500	46,200	63,200	28,100	32,100	37,000	70,800	13,800	29,700	46,400	40,800	30,417	21,500	30,900	35,900	36,800
MEDP0081	Serotonin	**177**	C10H12N2O	9	9	9	9	9	9	77,100	71,300	147,000	46,800	62,700	57,900	186,000	49,600	41,700	55,500	79,731	65,600	57,600	70,800	62,500
MEDP0082	S-Sulfo-L-Cysteine	**202.2**	C3H7NO5S2	9	9	9	9	9	9	17,800	25,800	17,200	17,900	17,700	10,700	20,800	14,100	12,700	12,200	14,271	11,500	26,900	23,300	28,800
MEDP0083	Trans-4-Hydroxy-L-Proline	**132.058**	C5H9NO3	32,200,000	34,000,000	33,100,000	33,200,000	33,100,000	34,300,000	12,700,000	18,300,000	12,300,000	11,500,000	9,750,000	11,800,000	7,250,000	7,470,000	5,010,000	7,690,000	6,522,520	5,190,000	12,100,000	13,400,000	14,400,000
MEDP0085	Tyramine	**138.084**	C8H11NO	143,000	37,300	40,900	175,000	61,400	260,000	1,120,000	154,000	3,170,000	1,730,000	157,000	399,000	41,700	86,100	76,300	78,900	68,415	59,100	428,000	424,000	479,000
MEDP0087	L-Alanyl-L-Lysine	**218.1**	C9H19N3O3	9	9	9	9	9	9	879,000	853,000	761,000	806,000	822,000	1,150,000	1,360,000	903,000	1,110,000	1,120,000	1,088,452	953,000	782,000	937,000	984,000
MEDP0089	N-Acetylhistamine	**154.1**	C7H11N3O	3,060,000	3,550,000	3,680,000	2,910,000	4,350,000	3,510,000	1,580,000	1,690,000	1,410,000	1,320,000	1,940,000	1,520,000	1,380,000	1,620,000	1,280,000	1,350,000	1,387,120	1,300,000	2,100,000	1,930,000	2,340,000
MEDP0101	P-Coumaric Acid	**165.047**	C9H8O3	7,150,000	7,460,000	7,310,000	7,030,000	7,490,000	7,190,000	16,900,000	17,000,000	17,600,000	16,000,000	17,400,000	16,700,000	12,100,000	13,800,000	13,600,000	13,200,000	13,170,000	13,100,000	13,800,000	13,800,000	13,900,000
MEDP0113	4-Hydroxybenzoic Acid	**139.03**	C7H6O3	424,000	136,000	63,900	92,100	232,000	314,000	252,000	320,000	169,000	117,000	214,000	442,000	679,000	528,000	537,000	607,000	590,748	602,000	311,000	358,000	386,000
MEDP0118	4-Pyridoxic Acid	**184.05**	C8H9NO4	469,000	265,000	264,000	288,000	396,000	466,000	638,000	833,000	411,000	372,000	701,000	876,000	812,000	649,000	524,000	706,000	670,204	660,000	766,000	733,000	782,000

MEDP0119	6-Hydroxynicotinic Acid	**140**	C6H5NO3	19,800	17,600	15,500	29,300	23,300	18,800	20,800	16,800	26,100	10,900	21,100	29,100	26,700	9	21,100	25,100	17,562	14,900	19,600	26,900	20,400
MEDP0125	Choline	**105.11**	C5H14NO+	231,000	271,000	250,000	267,000	306,000	287,000	1,090,000	1,120,000	1,020,000	1,240,000	1,030,000	1,030,000	1,940,000	1,640,000	1,650,000	1,850,000	1,774,280	1,790,000	1,570,000	1,630,000	1,550,000
MEDP0126	1,5-Diaminopentane	**103**	C5H14N2	33,200	15,700	39,800	166,000	60,500	45,900	34,800	22,800	37,400	27,300	41,200	45,500	14,300	17,300	13,300	22,300	15,682	11,300	29,400	36,500	22,900
MEDP0135	Myoinositol	**181.06**	C6H12O6	1,670,000	1,910,000	1,220,000	1,490,000	2,050,000	1,530,000	1,390,000	1,730,000	1,450,000	1,740,000	1,020,000	1,020,000	1,590,000	686,000	877,000	1,510,000	1,141,254	1,050,000	1,140,000	1,020,000	1,240,000
MEDP0143	Biotin	**245.09**	C10H16N2O3S	108,000	133,000	141,000	115,000	154,000	140,000	163,000	170,000	162,000	134,000	172,000	179,000	156,000	178,000	186,000	175,000	174,144	176,000	149,000	187,000	192,000
MEDP0147	1-Methylhistidine	**170.1**	C7H11N3O2	687,000	938,000	1,040,000	1,010,000	839,000	1,060,000	509,000	317,000	577,000	532,000	507,000	613,000	262,000	286,000	282,000	356,000	303,334	331,000	365,000	434,000	386,000
MEDP0148	1-Methylxanthine	**167.1**	C6H6N4O2	9	9	9	9	9	9	4,280	9	9	9	9	21,400	39,900	37,500	48,600	44,600	42,509	41,900	49,000	33,500	43,000
MEDP0149	2′-Deoxyadenosine-5′-Monophosphate	**332**	C10H14N5O6P	9	9	9	9	9	9	119,000	109,000	112,000	95,100	140,000	139,000	96,500	133,000	109,000	126,000	123,110	151,000	94,000	95,100	99,800
MEDP0150	2′-Deoxyinosine	**253.1**	C10H12N4O4	348,000	421,000	453,000	405,000	378,000	413,000	54,800	50,000	41,500	58,700	52,700	71,200	37,600	43,300	57,000	31,600	43,645	48,800	148,000	175,000	173,000
MEDP0152	3′-Aenylic Acid	**348**	C10H14N5O7P	2,270,000	1,870,000	1,860,000	1,840,000	2,110,000	2,050,000	453,000	465,000	356,000	527,000	365,000	555,000	69,100	87,500	57,500	75,600	67,173	46,200	503,000	556,000	541,000
MEDP0155	5-Methylcytosine	**126.1**	C5H7N3O	78,800	64,200	59,700	56,200	83,400	64,700	35,400	43,400	21,900	35,600	46,200	30,000	45,100	47,200	50,200	61,100	50,634	49,600	46,100	61,600	40,200
MEDP0156	5-Methyluridine	**259.085**	C10H14N2O6	67,600	73,800	64,900	65,800	64,900	54,300	48,300	41,900	52,700	35,600	54,700	56,700	46,500	61,200	60,500	55,400	59,143	72,200	52,500	42,200	83,900
MEDP0159	Adenine	**136.1**	C5H5N5	1,030,000	1,180,000	1,090,000	1,140,000	1,220,000	1,110,000	2,620,000	2,450,000	2,880,000	2,700,000	2,530,000	2,520,000	1,790,000	1,890,000	2,200,000	1,880,000	1,930,160	1,880,000	1,950,000	1,890,000	1,990,000

MEDP0160	Adenosine	**268.097**	C10H13N5O4	832,000	868,000	1,160,000	1,250,000	1,120,000	1,070,000	20,900,000	22,000,000	19,000,000	17,700,000	17,700,000	27,900,000	1,070,000	983,000	715,000	1,220,000	958,222	795,000	7,110,000	8,740,000	8,820,000
MEDP0163	Cytidine	**244.086**	C9H13N3O5	8,870,000	9,560,000	10,400,000	7,380,000	6,990,000	8,910,000	5,000,000	5,170,000	5,180,000	4,490,000	5,210,000	4,960,000	6,470,000	6,340,000	6,590,000	6,840,000	6,770,980	7,600,000	5,720,000	6,320,000	6,410,000
MEDP0164	Cytidine-5-Monophosphate	**324**	C9H14N3O8P	1,160,000	896,000	1,010,000	970,000	1,160,000	1,050,000	299,000	291,000	306,000	322,000	348,000	229,000	297,000	441,000	345,000	492,000	391,144	381,000	507,000	421,000	444,000
MEDP0165	Cytosine	**112**	C4H5N3O	2,010,000	2,400,000	2,180,000	1,760,000	2,140,000	2,470,000	1,110,000	1,440,000	1,050,000	907,000	1,000,000	1,150,000	1,310,000	1,510,000	945,000	1,100,000	1,221,540	1,240,000	1,670,000	1,380,000	1,300,000
MEDP0166	Deoxyguanosine	**268.097**	C10H13N5O4	9	9	9	9	9	9	72,800	71,400	70,100	64,700	73,100	84,700	133,000	145,000	139,000	168,000	146,318	146,000	89,500	93,000	94,000
MEDP0167	Guanosine	**284.092**	C10H13N5O5	4,440,000	4,170,000	4,720,000	5,110,000	4,280,000	4,910,000	40,400,000	43,700,000	40,800,000	35,400,000	39,200,000	43,200,000	37,700,000	44,100,000	42,000,000	48,800,000	43,480,400	44,800,000	34,900,000	36,700,000	36,500,000
MEDP0169	Guanosine Monophosphate	**364**	C10H14N5O8P	1,840,000	1,540,000	1,250,000	1,390,000	1,970,000	1,520,000	511,000	503,000	400,000	602,000	491,000	561,000	32,900	43,500	40,000	47,300	37,185	22,200	323,000	398,000	345,000
MEDP0170	Hypoxanthine	**137**	C5H4N4O	208,000,000	217,000,000	206,000,000	205,000,000	211,000,000	211,000,000	180,000,000	177,000,000	179,000,000	176,000,000	181,000,000	187,000,000	187,000,000	183,000,000	184,000,000	186,000,000	185,376,000	187,000,000	192,000,000	194,000,000	191,000,000
MEDP0171	Inosine	**269.1**	C10H12N4O5	192,000,000	151,000,000	138,000,000	145,000,000	185,000,000	159,000,000	429,000,000	444,000,000	424,000,000	438,000,000	426,000,000	410,000,000	474,000,000	420,000,000	420,000,000	398,000,000	429,790,000	437,000,000	375,000,000	397,000,000	407,000,000
MEDP0173	Nicotinic Acid Adenine Dinucleotide	**664.11**	C21H27N7O14P2	159,000	215,000	176,000	153,000	133,000	203,000	6,290	7,160	3,600	7,320	13,400	9	9	9	9	9	9	9	39,800	21,200	37,200
MEDP0174	Purine	**121**	C5H4N4	1,750,000	2,190,000	2,090,000	1,950,000	1,740,000	1,900,000	2,230,000	1,820,000	3,220,000	1,640,000	2,740,000	1,740,000	1,220,000	1,170,000	1,150,000	1,190,000	1,177,040	1,140,000	1,710,000	1,620,000	1,830,000
MEDP0175	Pyrimidinefreebase	**81.2**	C4H4N2	9	101,000	57,500	66,700	54,900	76,000	136,000	117,000	158,000	128,000	133,000	141,000	75,300	91,100	118,000	72,800	89,386	89,600	84,600	93,600	80,100

MEDP0176	Thymidine	**243.09**	C10H14N2O5	83,400	103,000	77,200	87,800	93,600	82,400	102,000	114,000	118,000	71,800	110,000	97,700	115,000	96,800	92,000	94,300	101,302	108,000	101,000	115,000	112,000
MEDP0177	Thymine	**127**	C5H6N2O2	199,000	236,000	262,000	273,000	236,000	226,000	88,000	45,300	110,000	88,300	69,700	127,000	160,000	149,000	156,000	155,000	163,088	196,000	133,000	190,000	129,000
MEDP0178	Uracil	**113.027**	C4H4N2O2	2,730,000	2,580,000	2,480,000	2,260,000	2,700,000	2,410,000	4,110,000	4,670,000	3,460,000	3,860,000	4,450,000	4,090,000	4,080,000	4,260,000	3,490,000	3,840,000	3,851,400	3,580,000	4,310,000	4,220,000	4,420,000
MEDP0179	Uridine	**245**	C9H12N2O6	429,000	329,000	353,000	292,000	408,000	321,000	338,000	390,000	305,000	235,000	363,000	395,000	269,000	305,000	322,000	302,000	283,130	217,000	319,000	321,000	327,000
MEDP0180	B-Nicotinamide Mononucleotide	**335.1**	C11H15N2O8P	4,650,000	4,770,000	5,060,000	4,850,000	4,580,000	4,850,000	388,000	351,000	430,000	400,000	391,000	371,000	173,000	194,000	187,000	204,000	187,162	178,000	734,000	712,000	714,000
MEDP0185	Epinephrine	**184**	C9H13NO3	433,000	564,000	564,000	491,000	449,000	476,000	1,560,000	1,280,000	1,550,000	1,820,000	1,470,000	1,690,000	1,590,000	1,350,000	1,190,000	1,500,000	1,386,480	1,300,000	1,160,000	1,030,000	1,000,000
MEDP0186	L-Thyroxine	**777.687**	C15H11I4NO4	38,100	45,500	45,200	55,400	41,700	45,800	46,000	43,300	45,000	39,400	45,000	57,300	36,100	47,000	49,100	43,900	42,487	36,400	47,000	50,100	53,800
MEDP0187	Metanephrine	**198.1**	C10H15NO3	9	9	9	9	9	9	24,100	18,700	24,800	19,800	33,600	23,900	31,100	29,600	25,300	14,700	27,486	36,700	19,000	9,520	20,400
MEDP0188	Norepinephrine	**170**	C8H11NO3	641,000	823,000	746,000	929,000	750,000	739,000	608,000	742,000	481,000	451,000	678,000	689,000	498,000	597,000	586,000	537,000	560,328	584,000	738,000	723,000	633,000
MEDP0208	Succinic Acid	**119.03**	C4H6O4	9	9	9	9	9	9	741,000	741,000	682,000	765,000	812,000	703,000	1,230,000	1,200,000	1,170,000	1,190,000	1,191,020	1,170,000	845,000	720,000	877,000
MEDP0224	D-Fructose	**181**	C6H12O6	12,500,000	11,500,000	7,460,000	9,780,000	10,200,000	9,190,000	8,320,000	10,500,000	9,290,000	9,040,000	6,420,000	6,330,000	9,370,000	4,750,000	4,170,000	7,810,000	6,412,340	5,950,000	6,540,000	6,930,000	7,190,000
MEDP0227	D-Mannose	**181**	C6H12O6	11,700,000	10,600,000	7,810,000	9,000,000	11,200,000	8,380,000	7,950,000	9,150,000	8,660,000	9,070,000	6,090,000	6,770,000	10,700,000	5,090,000	6,310,000	8,130,000	7,380,400	6,650,000	7,790,000	7,130,000	7,810,000
MEDP0229	Lactose	**343**	C12H22O11	47,100	30,000	7,910	13,000	13,600	10,300	138,000	122,000	155,000	127,000	154,000	132,000	14,100	20,300	12,400	29,800	19,131	19,100	78,900	70,300	72,000

MEDP0230	Maltose	**343**	C12H22O11	47,100	30,000	7,910	13,000	13,600	10,300	138,000	122,000	155,000	127,000	154,000	132,000	14,100	20,300	12,400	29,800	19,131	19,100	78,900	70,300	72,000
MEDP0232	N-Acetyl-D-Glucosamine	**222**	C8H15NO6	502,000	627,000	403,000	429,000	529,000	488,000	600,000	615,000	565,000	463,000	710,000	647,000	458,000	524,000	546,000	460,000	484,800	436,000	512,000	605,000	513,000
MEDP0239	L-Ascorbate	**177**	C6H8O6	212,000	198,000	354,000	167,000	287,000	171,000	226,000	267,000	319,000	223,000	133,000	189,000	202,000	117,000	143,000	203,000	198,224	326,000	194,000	216,000	214,000
MEDP0240	Pyridoxine	**170.1**	C8H11NO3	2,260,000	1,990,000	1,750,000	1,450,000	2,050,000	2,180,000	1,610,000	1,900,000	1,100,000	1,040,000	1,800,000	2,190,000	969,000	1,040,000	1,120,000	1,110,000	1,074,088	1,130,000	2,110,000	2,110,000	1,960,000
MEDP0242	Nicotinamide	**123.1**	C6H6N2O	142,000,000	129,000,000	129,000,000	125,000,000	133,000,000	136,000,000	258,000,000	250,000,000	268,000,000	253,000,000	256,000,000	263,000,000	196,000,000	195,000,000	203,000,000	189,000,000	197,350,000	202,000,000	204,000,000	206,000,000	203,000,000
MEDP0243	4-Oxoretinol	**301.2**	C20H28O2	9	9	9	9	9	9	54,600	85,300	25,200	24,100	89,000	49,400	40,500	9	9	74,600	25,954	14,700	34,400	42,500	27,100
MEDP0244	All-Trans-13,14-Dihydro-retinol	**287.2**	C20H32O	9	9	9	9	9	9	950,000	834,000	1,280,000	1,040,000	608,000	983,000	143,000	184,000	193,000	125,000	171,588	213,000	348,000	413,000	415,000
MEDP0246	Nicotinic Acid	**124**	C6H5NO2	1,200,000	411,000	600,000	2,050,000	1,360,000	939,000	9,140,000	6,230,000	12,600,000	7,520,000	7,880,000	11,500,000	10,900,000	12,000,000	11,700,000	13,400,000	12,083,600	12,500,000	8,640,000	8,280,000	8,230,000
MEDP0249	Pantothenol	**206.13**	C9H19NO4	17,600	24,100	18,500	22,900	11,100	9	10,700	18,900	21,400	13,300	9	9	73,300	68,200	83,800	87,000	80,099	88,100	37,000	41,900	38,000
MEDP0250	Riboflavin	**377**	C17H20N4O6	8,300,000	8,560,000	8,340,000	8,050,000	8,970,000	8,630,000	19,200,000	21,400,000	17,900,000	13,700,000	22,700,000	20,500,000	31,700,000	32,300,000	32,600,000	29,300,000	31,393,000	31,100,000	19,500,000	21,600,000	21,900,000
MEDP0251	Trigonelline	**138**	C7H7NO2	1,850,000	1,660,000	1,720,000	1,580,000	1,640,000	1,720,000	868,000	812,000	814,000	905,000	835,000	973,000	752,000	732,000	747,000	755,000	748,028	755,000	882,000	780,000	752,000
MEDP0271	3-Indolepropionic Acid	**190.08**	C11H11NO2	174,000	165,000	174,000	171,000	151,000	169,000	179,000	111,000	303,000	197,000	139,000	146,000	110,000	192,000	181,000	146,000	165,044	196,000	153,000	187,000	200,000

MEDP0272	5-Hydroxyindole-3-Acetic Acid	**192.06**	C10H9NO3	9	9	9	9	9	9	75,400	34,300	131,000	106,000	35,300	70,700	22,200	121,000	94,400	63,600	81,182	105,000	53,900	58,900	53,100
MEDP0275	Indole-3-Acetic Acid	**176.1**	C10H9NO2	3,390,000	3,430,000	3,440,000	3,310,000	3,440,000	3,580,000	5,480,000	5,490,000	5,830,000	5,260,000	5,170,000	5,670,000	3,510,000	4,530,000	4,400,000	4,280,000	4,190,140	4,230,000	4,660,000	4,580,000	4,430,000
MEDP0296	4-Guanidinobutyric Acid	**146.085**	C5H11N3O2	78,300,000	77,900,000	74,200,000	73,100,000	73,300,000	76,600,000	30,600,000	28,200,000	29,600,000	27,900,000	34,500,000	33,000,000	27,900,000	33,400,000	38,400,000	29,900,000	33,482,800	37,900,000	24,400,000	27,400,000	25,300,000
MEDP0297	5-Aminovaleric Acid	**118.2**	C5H11NO2	500,000	426,000	553,000	485,000	491,000	475,000	296,000	252,000	296,000	313,000	338,000	280,000	306,000	315,000	310,000	290,000	301,230	285,000	243,000	289,000	269,000
MEDP0298	6-Aminocaproic Acid	**132.094635**	C6H13NO2	348,000,000	373,000,000	368,000,000	349,000,000	366,000,000	347,000,000	523,000,000	520,000,000	501,000,000	541,000,000	524,000,000	527,000,000	544,000,000	518,000,000	510,000,000	545,000,000	525,366,000	510,000,000	506,000,000	512,000,000	515,000,000
MEDP0299	7-Methyluric Acid	**183**	C6H6N4O3	2,380,000	2,600,000	2,540,000	2,680,000	2,600,000	2,400,000	2,220,000	2,190,000	2,190,000	2,360,000	2,150,000	2,200,000	2,420,000	2,230,000	2,420,000	2,290,000	2,346,600	2,380,000	2,200,000	2,300,000	2,330,000
MEDP0300	Adipic Acid	**147.0579**	C6H10O4	9	9	9	9	9	9	20,600	18,800	19,600	24,200	13,900	26,800	71,900	61,500	51,200	80,900	70,432	86,700	37,600	36,200	32,100
MEDP0301	Azelaic Acid	**189.1049**	C9H16O4	717,000	598,000	601,000	666,000	678,000	678,000	986,000	1,390,000	692,000	711,000	944,000	1,190,000	3,220,000	2,680,000	2,430,000	3,400,000	2,887,280	2,710,000	1,630,000	1,560,000	1,630,000
MEDP0305	Creatinine	**114.0589**	C4H7N3O	7,920,000	7,700,000	7,700,000	7,460,000	8,740,000	8,090,000	22,700,000	21,100,000	21,300,000	17,000,000	29,100,000	25,100,000	46,000,000	54,700,000	59,600,000	51,200,000	54,452,000	60,800,000	21,100,000	22,900,000	23,200,000
MEDP0307	Dl-2-Aminooctanoic Acid	**160.126**	C8H17NO2	287,000	269,000	299,000	257,000	269,000	316,000	885,000	988,000	761,000	667,000	930,000	1,080,000	1,250,000	982,000	1,080,000	1,060,000	1,101,472	1,130,000	782,000	829,000	807,000
MEDP0313	Guanidineacetic Acid	**118.1**	C3H7N3O2	508,000	446,000	466,000	536,000	483,000	483,000	308,000	286,000	305,000	314,000	348,000	289,000	293,000	332,000	298,000	298,000	308,738	323,000	276,000	307,000	267,000
MEDP0315	Hippuric Acid	**180.1**	C9H9NO3	10,800,000	11,500,000	11,200,000	11,800,000	11,000,000	11,300,000	11,600,000	11,000,000	13,300,000	10,900,000	11,200,000	11,300,000	11,600,000	12,100,000	11,900,000	11,600,000	11,923,600	12,300,000	11,500,000	12,100,000	12,200,000

MEDP0318	Kinurenine	**209.1**	C10H12N2O3	787,000	819,000	817,000	811,000	804,000	808,000	860,000	729,000	974,000	731,000	870,000	996,000	619,000	644,000	616,000	600,000	620,278	623,000	722,000	751,000	798,000
MEDP0319	Kynurenic Acid	**190.0426**	C10H7NO3	106,000	101,000	98,800	105,000	89,800	105,000	201,000	198,000	200,000	164,000	196,000	251,000	100,000	146,000	190,000	143,000	143,296	137,000	184,000	151,000	160,000
MEDP0321	L-Dihydroorotic Acid	**159**	C5H6N2O4	18,100,000	15,500,000	15,600,000	18,300,000	15,000,000	16,100,000	14,200,000	13,900,000	15,100,000	15,100,000	13,200,000	13,600,000	15,100,000	13,700,000	15,900,000	14,200,000	14,630,200	14,200,000	13,800,000	16,400,000	13,200,000
MEDP0322	L-Homoserine	**120.1**	C4H9NO3	2,240,000	2,610,000	2,870,000	2,850,000	2,360,000	2,620,000	1,080,000	918,000	1,180,000	1,170,000	1,090,000	1,050,000	920,000	890,000	931,000	857,000	916,304	983,000	909,000	1,030,000	945,000
MEDP0325	Maleic Acid	**117.011**	C4H4O4	3,140,000	3,590,000	3,170,000	3,300,000	3,020,000	3,240,000	2,570,000	2,270,000	2,680,000	2,410,000	2,810,000	2,690,000	3,940,000	4,030,000	4,380,000	3,640,000	4,106,260	4,550,000	2,900,000	3,080,000	2,910,000
MEDP0326	N′-Formylkynurenine	**237.1**	C11H12N2O4	76,100	93,200	122,000	123,000	134,000	110,000	346,000	332,000	369,000	312,000	390,000	329,000	325,000	204,000	224,000	244,000	240,584	205,000	215,000	251,000	248,000
MEDP0331	Subericacid	**175.1**	C8H14O4	315,000	314,000	299,000	310,000	332,000	308,000	520,000	659,000	393,000	417,000	532,000	601,000	1,760,000	1,460,000	1,420,000	1,870,000	1,614,780	1,560,000	883,000	903,000	879,000
MEDP0332	Cinnamic Acid	**149.052**	C9H8O2	22,100	13,400	24,900	18,800	18,500	26,000	44,000	56,200	31,300	42,000	51,200	39,400	43,400	28,700	29,100	34,500	34,502	36,700	25,900	33,800	36,800
MEDP0357	Urocanic Acid	**139.04**	C6H6N2O2	9	85,000	73,000	96,300	9	9	61,100	35,800	60,800	96,300	55,000	57,700	75,500	58,500	73,600	83,000	68,962	54,200	65,000	77,100	65,800
MEDP0367	γ-Aminobutyric Acid	**104.06**	C4H9NO2	38,100	56,000	70,500	57,300	51,600	52,100	4,310	9	9	10,500	11,100	9	8,050	9	9	9	3,611	9,980	12,800	6,600	8,380
MEDP0368	Taurocholic Acid Sodium Salt Hydrate	**556.28**	C26H46NNaO8S	3,560,000	3,880,000	4,260,000	3,610,000	4,310,000	4,290,000	3,800,000	3,570,000	3,920,000	4,310,000	3,700,000	3,480,000	3,820,000	4,170,000	3,730,000	3,930,000	3,954,160	4,120,000	3,480,000	3,880,000	4,120,000
MEDP0370	2-(Formylamino)Benzoic Acid	**166.04**	C8H7NO3	72,300	96,700	97,600	107,000	84,300	98,700	164,000	178,000	157,000	137,000	155,000	196,000	415,000	368,000	353,000	380,000	382,688	398,000	229,000	254,000	233,000
MEDP0372	Uridine 5-Monophosphate	**325**	C9H13N2O9P	14,200	17,800	34,900	21,600	9	24,600	46,300	45,300	46,000	62,100	26,600	51,600	14,200	9	9	9	2,853	9	26,200	29,400	33,600

MEDP0373	N-Acetylglycine	**118**	C4H7NO3	124,000,000	123,000,000	121,000,000	121,000,000	118,000,000	119,000,000	56,200,000	51,200,000	54,900,000	51,600,000	63,100,000	60,000,000	38,800,000	44,000,000	52,500,000	41,000,000	45,611,200	51,800,000	42,700,000	47,500,000	45,800,000
MEDP0378	N6-Succinyl Adenosine	**384**	C14H17N5O8	4,200,000	5,400,000	5,810,000	4,890,000	5,070,000	5,050,000	1,620,000	1,530,000	1,470,000	1,200,000	2,000,000	1,920,000	2,740,000	3,080,000	2,810,000	2,580,000	2,849,460	3,040,000	3,090,000	3,180,000	3,500,000
MEDP0380	2-(Dimethylamino)Guanosine	**312**	C12H17N5O5	1,570,000	1,600,000	1,530,000	1,600,000	1,640,000	1,500,000	370,000	340,000	397,000	271,000	414,000	427,000	703,000	748,000	730,000	919,000	748,970	646,000	1,090,000	1,140,000	1,110,000
MEDP0383	B-Pseudouridine	**245**	C9H12N2O6	429,000	329,000	353,000	292,000	408,000	321,000	338,000	390,000	305,000	235,000	363,000	395,000	269,000	305,000	322,000	302,000	283,130	217,000	319,000	321,000	327,000
MEDP0387	L-Homoargininei	**189**	C7H16N4O2	2,690,000	3,440,000	3,550,000	3,310,000	2,900,000	3,350,000	299,000	314,000	255,000	187,000	371,000	369,000	144,000	183,000	196,000	183,000	179,040	190,000	255,000	335,000	316,000
MEDP0389	Pantetheine	**279.13**	C11H22N2O4S	13,800,000	15,000,000	14,700,000	16,000,000	17,000,000	16,900,000	21,900,000	23,300,000	16,200,000	15,400,000	19,600,000	35,200,000	13,800,000	13,300,000	12,700,000	16,500,000	13,965,000	13,600,000	15,400,000	17,400,000	16,600,000
MEDP0393	Pyridoxine 5′-Phosphate	**250**	C8H12NO6P	182,000	189,000	112,000	77,100	222,000	142,000	1,190,000	1,150,000	1,220,000	1,010,000	1,320,000	1,230,000	683,000	897,000	839,000	808,000	814,882	847,000	749,000	790,000	936,000
MEDP0395	DL-Pipecolic Acid	**130**	C6H11NO2	2,010,000	2,310,000	2,360,000	2,440,000	2,210,000	2,290,000	2,090,000	2,340,000	2,290,000	1,860,000	1,560,000	2,420,000	5,100,000	4,930,000	5,030,000	4,570,000	4,913,820	4,940,000	3,470,000	3,360,000	2,660,000
MEDP0396	2′-Deoxycytidine-5′-Monophosphate	**308**	C9H14N3O7P	9	9	9	9	9	9	368,000	318,000	389,000	328,000	357,000	451,000	693,000	981,000	985,000	795,000	884,910	971,000	415,000	414,000	465,000
MEDP0401	5′-Deoxy-5′-(Methylthio) Adenosine	**298**	C11H15N5O3S	796,000	924,000	1,240,000	1,340,000	903,000	1,070,000	92,500	102,000	80,300	80,500	100,000	99,000	9	9	9	9	9	9	238,000	258,000	275,000
MEDP0403	Deoxycytidine	**228**	C9H13N3O4	102,000	86,500	62,700	44,500	73,700	54,000	112,000	136,000	91,700	73,900	127,000	132,000	85,100	77,600	91,200	94,900	85,816	80,300	73,900	54,800	97,900
MEDP0409	Cys-Gly	**179**	C5H10N2O3S	1,230,000	1,290,000	1,340,000	1,230,000	1,220,000	1,330,000	40,400	13,500	83,300	53,800	20,800	30,400	6,490	24,700	11,400	9	9,291	3,920	137,000	144,000	180,000

MEDP0430	2-Aminoadipic Acid	**162.1**	C6H11NO4	2,590,000	3,390,000	3,740,000	3,230,000	2,720,000	2,820,000	164,000	126,000	192,000	154,000	193,000	156,000	188,000	177,000	256,000	180,000	194,506	171,000	348,000	351,000	369,000
MEDP0435	6-Methyl mercaptopurine	**167.1**	C6H6N4S	55,800,000	57,300,000	58,600,000	55,500,000	56,600,000	57,600,000	142,000,000	136,000,000	149,000,000	134,000,000	146,000,000	143,000,000	116,000,000	127,000,000	123,000,000	120,000,000	122,346,000	125,000,000	114,000,000	116,000,000	116,000,000
MEDP0442	Sn-Glycero-3-Phosphocho-line	**258.2**	C8H20NO6P	19,600,000	21,200,000	22,600,000	20,800,000	19,300,000	22,900,000	5,360,000	4,480,000	5,470,000	5,250,000	5,670,000	5,900,000	7,590,000	7,740,000	7,650,000	7,160,000	7,621,880	7,970,000	6,110,000	6,840,000	6,220,000
MEDP0446	3-Hydroxykynurenine	**225.1**	C10H12N2O4	136,000	241,000	227,000	175,000	166,000	184,000	28,200	34,400	9	29,700	47,000	30,100	51,800	53,600	24,200	45,400	41,132	30,600	64,000	52,600	80,900
MEDP0453	Indole	**118**	C8H7N	3,170,000	3,350,000	3,070,000	3,180,000	3,320,000	3,260,000	4,940,000	4,960,000	5,190,000	4,450,000	5,030,000	5,070,000	3,070,000	3,990,000	3,540,000	3,990,000	3,710,160	3,960,000	4,070,000	4,310,000	4,150,000
MEDP0457	5-Aminolevulinate	**132.1**	C5H9NO3	1,120,000	1,170,000	1,150,000	1,080,000	1,160,000	1,110,000	3,470,000	3,420,000	3,500,000	3,140,000	3,790,000	3,490,000	3,050,000	2,950,000	2,990,000	3,050,000	3,017,340	3,040,000	2,920,000	2,810,000	2,740,000
MEDP0461	2-Methylguanosine	**298.1**	C11H15N5O5	1,250,000	1,310,000	1,270,000	1,260,000	1,350,000	1,330,000	591,000	577,000	578,000	465,000	693,000	641,000	1,080,000	1,070,000	1,000,000	1,170,000	1,068,300	1,030,000	1,140,000	1,140,000	1,110,000
MEDP0504	Glucosamine	**180**	C6H13NO5	714,000	1,240,000	994,000	767,000	874,000	874,000	700,000	931,000	656,000	633,000	605,000	674,000	905,000	495,000	744,000	628,000	663,382	546,000	488,000	529,000	690,000
MEDP0507	2-Hydroxycinnamic acid	**165.047**	C9H8O3	7,150,000	7,460,000	7,310,000	7,030,000	7,490,000	7,190,000	16,900,000	17,000,000	17,600,000	16,000,000	17,400,000	16,700,000	12,100,000	13,800,000	13,600,000	13,200,000	13,170,000	13,100,000	13,800,000	13,800,000	13,900,000
MEDP0518	DL-Stachydrine	**144.1025**	C7H14ClNO2	1,910,000	2,390,000	2,150,000	1,480,000	1,550,000	1,570,000	795,000	771,000	889,000	655,000	820,000	840,000	957,000	839,000	877,000	834,000	883,704	912,000	669,000	780,000	733,000
MEDP0519	L-Norleucine	**132.1023**	C6H13NO2	5,640,000	5,590,000	5,430,000	5,350,000	5,480,000	5,750,000	10,700,000	9,980,000	11,500,000	9,790,000	10,800,000	11,600,000	6,590,000	9,370,000	8,980,000	8,620,000	8,456,480	8,730,000	8,380,000	8,970,000	8,590,000
MEDP0525	N-Acetyl-L-alanine	**132.058244**	C5H9NO3	17,200,000	19,700,000	19,500,000	17,700,000	18,800,000	17,300,000	51,400,000	50,100,000	53,000,000	51,600,000	54,200,000	48,000,000	44,200,000	49,300,000	45,000,000	45,900,000	45,803,200	44,700,000	44,300,000	43,100,000	44,400,000

MEDP0540	7,8-dihydro-L-Biopterin	**240.10184**	C9H13N5O3	9	9	9	9	9	9	139,000	136,000	137,000	136,000	129,000	159,000	96,400	95,800	101,000	74,300	93,790	102,000	68,500	99,700	73,100
MEDP0575	5-amino-1-[3,4-dihydroxy-5-(hydroxymethyl)oxolan-2-yl]imidazole-4-carbox-amide	**259.096421**	C9H14N4O5	1,770,000	2,190,000	2,750,000	2,750,000	2,040,000	2,300,000	2,580,000	2,450,000	2,680,000	2,230,000	2,620,000	2,910,000	1,180,000	2,060,000	1,760,000	1,490,000	1,658,200	1,800,000	2,210,000	2,140,000	2,290,000
MEDP0576	N1-Acetylspermine	**245.2263115**	C12H28N4O	9	9	9	9	9	9	24,000	23,300	20,400	9	28,300	48,200	127,000	99,200	112,000	115,000	114,833	120,000	32,100	39,900	52,100
MEDP0586	N-Acetylphenylalanine	**208.0895433**	C11H13NO3	47,200	61,800	61,900	65,500	62,900	64,500	688,000	542,000	552,000	420,000	823,000	1,100,000	1,340,000	1,240,000	1,270,000	1,190,000	1,263,560	1,270,000	610,000	686,000	692,000
MEDP0587	N-Alpha-acetyllysine	**189.1160924**	C8H16N2O3	26,500,000	37,500,000	38,600,000	36,300,000	28,100,000	33,900,000	4,930,000	3,460,000	4,250,000	2,600,000	6,710,000	7,640,000	1,650,000	3,120,000	2,680,000	1,790,000	2,379,140	2,650,000	4,010,000	3,970,000	3,980,000
MEDP0601	Spermidine	**146.1578976**	C7H19N3	1,580,000	1,130,000	1,060,000	1,070,000	1,320,000	1,080,000	10,100,000	9,370,000	11,000,000	8,410,000	11,300,000	10,600,000	9,940,000	12,100,000	13,900,000	11,400,000	12,029,900	12,800,000	7,260,000	7,820,000	8,020,000
MEDP0611	N-Acetyl-L-Histidine	**198.0800412**	C8H11N3O3	41,100,000	34,600,000	32,000,000	35,100,000	43,200,000	40,800,000	18,500,000	15,300,000	19,300,000	20,500,000	18,500,000	19,200,000	12,100,000	11,500,000	11,600,000	12,100,000	11,943,400	12,400,000	13,600,000	15,600,000	14,600,000
MEDP0627	Isonicotinic acid	**124.0320284**	C6H5NO2	54,400,000	47,300,000	49,900,000	48,400,000	51,400,000	52,800,000	115,000,000	113,000,000	122,000,000	109,000,000	114,000,000	118,000,000	78,500,000	80,200,000	84,000,000	75,300,000	80,333,000	83,600,000	82,600,000	86,800,000	83,100,000
MEDP0637	L-phenylalanyl-L-proline	**263.131743**	C14H18N2O3	1,210,000	1,290,000	1,440,000	1,370,000	1,250,000	1,370,000	20,300,000	18,400,000	23,700,000	21,900,000	19,300,000	18,000,000	16,700,000	22,900,000	18,500,000	21,800,000	20,544,800	22,900,000	15,300,000	15,400,000	15,300,000
MEDP0685	Methylcysteine	**136.2**	C4H9NO2S	7,310,000	7,300,000	7,590,000	7,310,000	7,690,000	7,460,000	17,500,000	17,900,000	17,700,000	16,500,000	18,500,000	17,100,000	13,100,000	14,500,000	15,600,000	13,900,000	14,232,000	14,200,000	14,000,000	14,800,000	14,500,000
MEDP0689	TranexamicAcid	**158.1**	C8H15NO2	55,000	65,100	52,100	74,600	63,900	61,500	65,900	75,100	65,200	46,500	69,700	73,100	56,600	50,500	51,900	65,000	58,715	69,600	64,900	51,700	45,100
MEDP0700	Methyl beta-D-galactopy-ranoside	**195.2**	C7H14O6	204,000	174,000	164,000	196,000	142,000	184,000	176,000	240,000	135,000	175,000	149,000	179,000	114,000	109,000	144,000	119,000	117,016	99,400	115,000	90,400	65,900

MEDP0729	Barbituric acid	**129**	C4H4N2O3	5,850,000	5,440,000	6,090,000	5,890,000	4,370,000	5,690,000	6,570,000	7,530,000	6,640,000	5,780,000	6,670,000	6,230,000	6,060,000	4,870,000	5,700,000	5,490,000	5,428,720	5,020,000	5,470,000	5,490,000	3,940,000
MEDP0752	DL-Leucine	**132.094635**	C6H13NO2	348,000,000	373,000,000	368,000,000	349,000,000	366,000,000	347,000,000	523,000,000	520,000,000	501,000,000	541,000,000	524,000,000	527,000,000	544,000,000	518,000,000	510,000,000	545,000,000	525,366,000	510,000,000	506,000,000	512,000,000	515,000,000
MEDP0821	6-Methylnicotinamide	**137.1**	C7H8N2O	71,100,000	58,000,000	49,400,000	54,300,000	66,600,000	58,100,000	244,000,000	242,000,000	242,000,000	249,000,000	242,000,000	245,000,000	234,000,000	229,000,000	210,000,000	238,000,000	226,596,000	223,000,000	200,000,000	208,000,000	194,000,000
MEDP0846	4-Methylbenzoic acid	**136.9**	C8H8O2	9	9	9	9	9	9	9	9	9	9	9	9	169,000	75,200	203,000	150,000	141,032	108,000	62,600	67,000	49,300
MEDP0849	Nα-Acetyl-L-glutamine	**189.1**	C7H12N2O4	33,600,000	46,100,000	47,700,000	44,800,000	36,900,000	42,700,000	3,500,000	2,790,000	2,800,000	2,290,000	4,720,000	4,910,000	2,270,000	2,770,000	3,260,000	2,610,000	2,787,260	3,030,000	5,190,000	5,370,000	5,240,000
MEDP0859	Glycyl-DL-phenylalanine	**223.2**	C11H14N2O3	6,350,000	5,640,000	5,490,000	5,860,000	6,460,000	6,420,000	140,000,000	146,000,000	139,000,000	133,000,000	138,000,000	144,000,000	88,000,000	74,100,000	76,500,000	76,400,000	79,335,000	81,600,000	81,500,000	87,900,000	87,900,000
MEDP0868	N-Methylalanine	**104**	C4H9NO2	1,370,000	1,630,000	1,520,000	1,660,000	1,550,000	1,500,000	5,320,000	5,830,000	4,990,000	6,040,000	4,940,000	4,810,000	7,820,000	7,880,000	7,900,000	8,920,000	8,247,660	8,720,000	7,390,000	7,430,000	7,350,000
MEDP0876	O-Succinyl-L-Homoserine	**220.2**	C8H13NO6	648,000	758,000	763,000	563,000	798,000	733,000	134,000	130,000	107,000	138,000	137,000	159,000	348,000	384,000	328,000	377,000	359,314	359,000	380,000	354,000	334,000
MEDP0878	N-Methyl-L-Glutamate	**162.3**	C6H11NO4	2,310,000	2,810,000	3,230,000	2,750,000	2,470,000	2,810,000	203,000	175,000	173,000	167,000	259,000	242,000	182,000	243,000	198,000	226,000	226,578	284,000	319,000	333,000	346,000
MEDP0879	N-Methyl-D-Aspartic Acid	**148.2**	C5H9NO4	9	9	9	9	9	9	95,800	72,200	94,900	112,000	137,000	63,200	105,000	80,700	104,000	132,000	109,801	127,000	161,000	138,000	186,000
MEDP0880	N-Alpha-Acetyl-L-Asparagine	**175.3**	C6H10N2O4	9	9	9	9	9	9	316,000	454,000	9	229,000	515,000	380,000	458,000	307,000	303,000	503,000	391,574	386,000	339,000	299,000	424,000

MEDP0881	Phosphocholine	**185.1**	$C_5H_{15}NO_4P$	16,500,000	15,900,000	15,300,000	14,400,000	16,000,000	15,000,000	15,600,000	16,700,000	14,500,000	13,700,000	15,900,000	17,200,000	13,000,000	13,000,000	11,700,000	12,600,000	12,368,000	11,400,000	13,800,000	14,900,000	14,300,000
MEDP0883	2,4-Dihydroxypteridine	**165.3**	$C_6H_4N_4O_2$	79,200	99,600	76,700	63,100	68,700	90,200	196,000	188,000	210,000	185,000	223,000	176,000	163,000	156,000	159,000	144,000	160,894	183,000	159,000	181,000	164,000
MEDP0884	L-Methionine Sulfoximine	**181.3**	$C_5H_{12}N_2O_3S$	20,000	14,300	17,400	11,300	6,530	9,790	38,600	33,900	50,400	52,100	36,600	19,800	66,100	70,800	73,100	84,400	69,771	54,400	43,500	44,000	60,900
MEDP0886	N-Amidino-L-Aspartate	**176.4**	$C_5H_9N_3O_4$	89,000	102,000	149,000	140,000	123,000	126,000	168,000	165,000	174,000	167,000	163,000	170,000	51,600	75,100	66,200	60,500	63,491	64,100	86,700	97,900	83,300
MEDP0889	Cortisol	**363.5**	$C_{21}H_{30}O_5$	34,300	38,700	41,100	35,400	36,600	36,800	43,100	37,600	39,300	41,900	49,100	47,500	33,800	29,500	32,800	35,500	33,276	34,800	36,700	39,600	41,600
MEDP0890	7,8-Dimethylalloxazine	**243.2**	$C_{12}H_{10}N_4O_2$	188,000	218,000	190,000	201,000	211,000	232,000	351,000	388,000	328,000	259,000	384,000	398,000	550,000	517,000	548,000	521,000	552,406	627,000	336,000	381,000	389,000
MEDP0891	L-Tryptophanamide	**204.3**	$C_{11}H_{13}N_3O$	189,000	171,000	184,000	165,000	193,000	180,000	304,000	289,000	311,000	258,000	323,000	338,000	225,000	274,000	251,000	260,000	252,082	251,000	228,000	269,000	276,000
MEDP0899	3-Methyladenine	**150.3**	$C_6H_7N_5$	57,100	97,600	68,300	72,700	76,500	87,000	33,200	23,000	28,200	36,000	36,100	42,600	33,400	35,000	43,700	30,800	32,009	17,200	32,500	32,600	32,500
MEDP1002	Acetylcholine	**146.1084**	$C_7H_{15}NO_2$	11,900,000	11,500,000	11,400,000	11,100,000	11,200,000	11,100,000	3,980,000	3,880,000	4,160,000	4,590,000	3,700,000	3,540,000	4,260,000	3,880,000	3,710,000	3,240,000	3,769,000	3,760,000	4,630,000	4,680,000	4,480,000
MEDP1005	β-Alanine	**90.048**	$C_3H_7NO_2$	37,500,000	37,800,000	38,700,000	37,100,000	36,300,000	38,400,000	8,070,000	10,200,000	6,810,000	6,290,000	7,890,000	9,140,000	4,920,000	6,300,000	5,800,000	5,830,000	5,797,060	6,150,000	8,020,000	7,910,000	7,900,000
MEDP1006	Creatine	**132.0671941**	$C_4H_9N_3O_2$	138,000,000	131,000,000	137,000,000	133,000,000	131,000,000	134,000,000	41,100,000	53,300,000	32,800,000	31,700,000	39,900,000	47,900,000	22,500,000	29,500,000	27,600,000	29,100,000	27,612,800	29,300,000	39,300,000	40,200,000	38,600,000
MEDP1010	Coenzyme II (β-NADP)	**744**	$C_{21}H_{28}N_7O_{17}P_3$	9	9	9	9	9	9	27,900	31,500	26,500	18,200	38,000	25,100	31,200	43,100	34,300	46,100	41,251	51,500	32,400	41,000	34,200

MEDP1018	5′-Deoxyadenosine	**252**	C10H13N5O3	70,300	113,000	109,000	96,300	99,100	108,000	25,600	25,000	22,300	24,200	23,800	32,700	32,200	44,900	37,700	37,800	38,348	39,100	42,600	44,400	62,400
MEDP1019	aminoisobutyric acid	**104**	C4H9NO2	1,370,000	1,630,000	1,520,000	1,660,000	1,550,000	1,500,000	5,320,000	5,830,000	4,990,000	6,040,000	4,940,000	4,810,000	7,820,000	7,880,000	7,900,000	8,920,000	8,247,660	8,720,000	7,390,000	7,430,000	7,350,000
MEDP1022	Threoninyl-Phenylalanine	**267.1243321**	C13H18N2O4	170,000	61,400	35,200	74,600	136,000	116,000	1,720,000	1,890,000	1,550,000	1,680,000	1,660,000	1,820,000	1,070,000	1,540,000	1,370,000	1,190,000	1,326,600	1,460,000	1,190,000	1,170,000	1,160,000
MEDP1023	Tryptophan glutamic acid	**334.1303767**	C16H19N3O5	57,000	62,100	81,800	68,500	67,400	69,000	2,250,000	2,010,000	2,360,000	1,910,000	2,300,000	2,670,000	1,830,000	2,930,000	2,720,000	2,490,000	2,587,060	2,960,000	1,330,000	1,520,000	1,600,000
MEDP1024	Valyl-leucine	**231.1606614**	C11H22N2O3	3,260,000	519,000	352,000	479,000	2,120,000	1,300,000	43,500,000	53,400,000	37,000,000	41,900,000	49,100,000	35,900,000	45,200,000	24,900,000	26,800,000	27,300,000	30,453,000	28,100,000	28,400,000	29,200,000	28,900,000
MEDP1025	L-Valyl-L-phenylalanine	**265.1453447**	C14H20N2O3	370,000	100,000	73,500	98,800	262,000	190,000	19,800,000	24,300,000	17,300,000	17,900,000	20,800,000	19,000,000	18,700,000	18,400,000	18,100,000	19,700,000	18,697,200	18,500,000	15,500,000	16,000,000	15,800,000
MEDP1045	Biliverdin	**583.2**	C33H34N4O6	13,100	5,130	6,020	7,280	7,530	8,260	10,900	21,300	9	9	15,500	17,500	11,500	9	9	9	2,616	1,570	7,520	4,710	10,300
MEDP1059	2-Picolinic Acid	**124.0839**	C6H5NO2	85,600	39,600	40,600	168,000	143,000	86,400	770,000	487,000	1,020,000	680,000	663,000	1,000,000	921,000	1,010,000	1,010,000	1,210,000	1,048,516	1,090,000	713,000	708,000	714,000
MEDP1061	DL-O-tyrosine	**166**	C9H11NO2	349,000,000	340,000,000	348,000,000	349,000,000	347,000,000	350,000,000	349,000,000	348,000,000	348,000,000	349,000,000	349,000,000	353,000,000	357,000,000	353,000,000	355,000,000	351,000,000	354,272,000	356,000,000	349,000,000	355,000,000	352,000,000
MEDP1063	Indole-5-carboxylic acid	**162.0452919**	C9H7NO2	9	9	9	9	9	9	122,000	219,000	25,100	36,700	204,000	126,000	358,000	129,000	244,000	288,000	243,918	200,000	128,000	121,000	125,000
MEDP1070	Glycyl-L-valine	**175.1**	C7H14N2O3	9	9	9	9	9	9	49,900	48,300	55,200	45,500	53,100	47,500	51,700	70,400	53,600	71,200	62,850	67,400	60,100	52,300	50,200
MEDP1071	Alanyl-dl-leucine	**203.1288496**	C9H18N2O3	437,000	177,000	120,000	175,000	384,000	309,000	12,800,000	14,800,000	10,900,000	11,500,000	14,500,000	12,400,000	21,500,000	27,400,000	25,300,000	23,200,000	24,807,400	26,700,000	13,700,000	14,300,000	14,300,000

MEDP1072	7-methylguanosine	**298.1**	C11H15N5O5	1,250,000	1,310,000	1,270,000	1,260,000	1,350,000	1,330,000	591,000	577,000	578,000	465,000	693,000	641,000	1,080,000	1,070,000	1,000,000	1,170,000	1,068,300	1,030,000	1,140,000	1,140,000	1,110,000
MEDP1073	6-O-methylguanine	**166.06296**	C6H7N5O	219,000	249,000	236,000	216,000	244,000	253,000	66,800	95,800	51,100	9	84,300	103,000	72,400	101,000	64,300	118,000	85,691	73,000	131,000	129,000	143,000
MEDP1077	2′-O-methyladenosine	**348.1**	C11H15N5O4	2,390,000	2,090,000	1,960,000	2,050,000	2,090,000	2,200,000	515,000	539,000	434,000	675,000	349,000	576,000	161,000	133,000	127,000	135,000	126,075	74,800	652,000	641,000	491,000
MEDP1080	Glycyl-tryptophan	**262.1094522**	C13H15N3O3	29,500	27,200	28,500	33,500	34,400	32,200	662,000	584,000	789,000	598,000	582,000	756,000	396,000	736,000	651,000	570,000	610,360	699,000	389,000	428,000	448,000
MEDP1083	Oxypurinol	**153**	C5H4N4O2	34,700,000	37,600,000	38,100,000	31,700,000	44,400,000	36,300,000	15,700,000	17,000,000	14,300,000	12,600,000	18,400,000	16,500,000	13,800,000	16,000,000	12,500,000	14,700,000	14,286,000	14,500,000	21,600,000	20,900,000	23,400,000
MEDP1093	Allopurinol	**137.0481928**	C5H4N4O	62,300,000	49,000,000	44,600,000	46,900,000	57,600,000	51,600,000	216,000,000	219,000,000	210,000,000	219,000,000	219,000,000	214,000,000	199,000,000	205,000,000	201,000,000	208,000,000	202,520,000	199,000,000	190,000,000	189,000,000	187,000,000
MEDP1106	Lithocholic acid	**359.2949615**	C24H40O3	9	9	9	9	9	9	121,000	84,300	148,000	115,000	90,700	166,000	14,900	77,900	62,200	52,900	53,588	60,200	51,900	53,500	50,700
MEDP1109	Glycocholic Acid	**466.3055321**	C26H43NO6	77,500	95,400	83,700	100,000	90,600	86,100	150,000	131,000	180,000	133,000	140,000	163,000	105,000	124,000	149,000	220,000	146,520	134,000	135,000	139,000	121,000
MEDP1126	(R)-(-)-2-phenylglycine	**120.0726939**	C8H11NO	18,300,000	18,200,000	17,900,000	17,200,000	17,400,000	17,900,000	47,800,000	46,500,000	50,400,000	44,600,000	48,900,000	48,400,000	37,600,000	43,900,000	41,400,000	40,500,000	41,028,200	41,700,000	41,500,000	38,000,000	38,500,000
MEDP1127	1-acetylindole	**160.1**	C10H9NO	949,000	998,000	952,000	965,000	770,000	825,000	1,100,000	1,030,000	1,260,000	1,050,000	1,040,000	1,120,000	814,000	899,000	959,000	859,000	889,786	917,000	1,100,000	1,020,000	944,000
MEDP1131	Arachidyl glycine	**352.31663**	C22H43NO3	36,400	42,800	27,900	56,300	20,700	121,000	51,800	47,100	60,800	39,500	69,500	42,000	100,000	72,200	24,400	44,000	59,041	54,300	11,600	22,100	19,000
MEDP1132	3-Carboxypropyltrimethylammonium	**146.1084**	C7H15NO2	11,900,000	11,500,000	11,400,000	11,100,000	11,200,000	11,100,000	3,980,000	3,880,000	4,160,000	4,590,000	3,700,000	3,540,000	4,260,000	3,880,000	3,710,000	3,240,000	3,769,000	3,760,000	4,630,000	4,680,000	4,480,000

MEDP1144	L-Isoleucine	**132.09222**	C6H13NO2	341,000,000	363,000,000	349,000,000	341,000,000	345,000,000	344,000,000	535,000,000	531,000,000	532,000,000	530,000,000	539,000,000	543,000,000	552,000,000	532,000,000	525,000,000	553,000,000	538,370,000	531,000,000	512,000,000	522,000,000	526,000,000
MEDP1161	Creatine phosphate	**212.0425549**	C4H10N3O5P	1,790,000	1,720,000	1,810,000	1,790,000	1,910,000	2,180,000	2,010,000	1,900,000	1,880,000	1,910,000	2,000,000	2,390,000	1,700,000	1,850,000	1,730,000	1,660,000	1,736,700	1,740,000	1,230,000	1,780,000	2,020,000
MEDP1162	1-Methylguanine	**166.07051**	C6H7N5O	547,000	496,000	576,000	522,000	544,000	543,000	267,000	254,000	270,000	202,000	278,000	330,000	323,000	299,000	286,000	353,000	314,072	309,000	383,000	377,000	342,000
MEDP1177	N-Methyl-α-aminoisobutyric acid	**235.2**	C5H11NO2	7,000,000	6,290,000	7,780,000	7,360,000	7,590,000	7,630,000	1,170,000	1,010,000	1,190,000	1,440,000	1,150,000	1,060,000	553,000	453,000	560,000	432,000	520,442	604,000	1,060,000	1,370,000	1,200,000
MEDP1194	Calcitriol	**417.3**	C27H44O3	47,100	59,100	44,000	35,700	60,000	48,400	22,800	24,200	10,300	37,400	24,700	17,500	10,300	9	9	9	2,065	9	17,800	22,800	23,000
MEDP1196	Biotinamide	**244.1082639**	C10H17N3O2S	15,600,000	17,200,000	15,900,000	14,900,000	15,800,000	15,600,000	10,900,000	10,000,000	11,600,000	9,930,000	10,800,000	12,100,000	15,900,000	16,600,000	17,100,000	15,900,000	16,647,400	17,700,000	13,800,000	14,700,000	15,000,000
MEDP1197	Hypotaurocyamine	**152**	C3H9N3O2S	581,000	524,000	577,000	689,000	490,000	664,000	5,060,000	5,300,000	4,950,000	4,640,000	4,960,000	5,470,000	5,130,000	5,780,000	5,510,000	6,040,000	5,579,140	5,440,000	4,440,000	4,660,000	4,530,000
MEDP1202	5′-deoxy-5′-fluoroadenosine	**270.1**	C10H12FN5O3	9	9	9	9	9	9	126,000	133,000	98,400	115,000	123,000	161,000	9	9	9	9	9	9	65,800	44,800	49,900
MEDP1222	1-Aminocyclohexanoic acid	**144.1**	C7H13NO2	11,100,000	9,790,000	10,500,000	10,000,000	10,100,000	10,400,000	3,170,000	2,750,000	3,440,000	3,820,000	2,760,000	3,090,000	3,700,000	3,560,000	2,810,000	3,030,000	3,277,040	3,290,000	4,120,000	4,230,000	3,990,000
MEDP1242	4-tert-butylbenzoic acid	**179.10617**	C11H14O2	249,000	239,000	228,000	232,000	267,000	267,000	241,000	250,000	259,000	238,000	196,000	264,000	300,000	238,000	248,000	256,000	267,466	295,000	138,000	161,000	151,000
MEDP1246	2,4-diacetamino-2,4,6-triphenoxy-D-mannopyranose	**247.1**	C10H18N2O5	5,590,000	5,240,000	5,010,000	4,490,000	5,930,000	5,660,000	150,000,000	142,000,000	163,000,000	146,000,000	150,000,000	148,000,000	109,000,000	126,000,000	122,000,000	117,000,000	119,438,000	124,000,000	98,600,000	103,000,000	101,000,000

MEDP1272	20,26-dihydroxyecdysone	**497.3**	C27H44O8	41,600,000	53,700,000	57,800,000	54,500,000	50,200,000	56,500,000	305,000,000	304,000,000	317,000,000	297,000,000	304,000,000	301,000,000	323,000,000	346,000,000	351,000,000	310,000,000	333,210,000	336,000,000	304,000,000	304,000,000	306,000,000
MEDP1294	L-Leucyl-L-glycine	**189.1143956**	C8H16N2O3	186,000	249,000	247,000	173,000	257,000	198,000	2,660,000	2,420,000	3,020,000	2,620,000	2,670,000	2,580,000	2,570,000	3,550,000	3,100,000	3,040,000	3,055,460	3,010,000	2,240,000	2,140,000	2,160,000
MEDP1295	Cytarabine	**244.0826219**	C9H13N3O5	8,990,000	10,000,000	10,300,000	7,330,000	7,870,000	9,510,000	4,250,000	5,110,000	3,680,000	3,520,000	4,310,000	4,650,000	5,060,000	5,660,000	4,850,000	4,430,000	4,942,500	4,720,000	5,280,000	5,330,000	5,300,000
MEDP1354	Mycosporine-serinol	**262.1278734**	C11H19NO6	9	9	9	9	9	9	56,400	56,900	75,300	45,600	49,700	54,700	82,300	85,100	72,300	66,700	77,269	79,900	53,900	63,500	55,900
MEDP1360	Mycosporine glycine	**246.1**	C10H15NO6	9	9	9	9	9	9	48,100	37,300	47,300	47,600	60,900	47,300	36,600	48,000	45,700	44,500	39,883	24,600	38,800	47,300	15,700
MEDP1467	5-Methyl-2′-deoxycytidine	**242**	C10H15N3O4	1,180,000	1,140,000	1,100,000	1,040,000	1,160,000	1,200,000	397,000	411,000	288,000	505,000	327,000	453,000	165,000	166,000	159,000	160,000	157,744	139,000	303,000	241,000	356,000
MEDP1468	D-panthenol	**206**	C9H19NO4	11,500	8,250	7,530	6,980	9,740	9	2,590	9	9	9	9	12,900	30,800	38,600	30,000	44,600	36,434	38,200	15,300	21,300	19,700
MEDP1480	Anserine	**241**	C10H16N4O3	68,800,000	61,000,000	59,600,000	59,800,000	64,900,000	65,000,000	26,700,000	22,000,000	28,800,000	30,500,000	25,400,000	26,800,000	15,300,000	13,500,000	13,700,000	13,600,000	14,039,200	14,200,000	20,100,000	21,000,000	21,200,000
MEDP1481	Menadiol Diacetate	**259**	C15H14O4	31,100	69,600	60,200	73,300	78,300	54,500	59,900	73,400	43,000	49,100	79,000	55,100	63,900	71,200	74,300	123,000	76,730	51,300	71,600	74,800	47,200
MEDP1488	SDMA	**203**	C8H18N4O2	1,910,000	2,150,000	2,070,000	2,170,000	2,070,000	2,090,000	2,570,000	2,580,000	2,890,000	2,410,000	2,410,000	2,550,000	1,450,000	1,630,000	1,600,000	1,480,000	1,526,160	1,480,000	1,960,000	1,720,000	1,670,000
MEDP1490	Cis-4-Hydroxy-D-Proline	**132**	C5H9NO3	3,450,000	3,550,000	3,460,000	3,390,000	3,450,000	3,260,000	718,000	942,000	599,000	589,000	654,000	807,000	570,000	493,000	439,000	529,000	502,974	484,000	1,340,000	962,000	918,000
MEDP1496	L-2-Aminobutyric acid	**104**	C4H9NO2	4,170,000	4,660,000	4,880,000	5,100,000	4,900,000	5,050,000	19,800,000	22,500,000	19,600,000	21,100,000	17,900,000	18,100,000	34,700,000	31,600,000	32,200,000	33,300,000	32,999,400	33,200,000	25,300,000	23,300,000	25,600,000

MEDP1498	8-Azaguanine	**153**	C4H4N6O	15,600,000	15,300,000	16,600,000	16,100,000	17,500,000	15,700,000	5,930,000	5,360,000	6,440,000	4,910,000	6,140,000	6,810,000	4,550,000	5,060,000	4,410,000	4,830,000	4,768,300	4,990,000	6,460,000	6,410,000	6,680,000
MEDP1501	Isocytosine	**112**	C4H5N3O	2,220,000	2,060,000	2,630,000	1,540,000	1,840,000	2,530,000	920,000	836,000	999,000	897,000	921,000	946,000	1,400,000	1,400,000	1,110,000	1,160,000	1,266,880	1,280,000	1,320,000	1,160,000	1,250,000
MEDP1506	Leu-Val	**231**	C11H22N2O3	351,000	221,000	115,000	176,000	300,000	284,000	739,000	808,000	632,000	721,000	946,000	588,000	1,220,000	323,000	463,000	501,000	594,992	464,000	565,000	556,000	541,000
MEDP1507	Phe-Asn	**280**	C13H17N3O4	9	9	9	9	9	9	1,310,000	1,130,000	1,380,000	1,260,000	1,460,000	1,310,000	1,870,000	1,310,000	1,400,000	1,410,000	1,469,580	1,360,000	1,030,000	1,090,000	929,000
MEDP1508	Phe-Met	**297**	C14H20N2O3S	9	9	9	9	9	9	3,420,000	3,150,000	3,610,000	3,990,000	2,990,000	3,360,000	1,820,000	1,820,000	1,540,000	1,420,000	1,701,620	1,900,000	1,930,000	1,990,000	2,020,000
MEDP1509	Glu-Met	**279**	C10H18N2O5S	242,000	292,000	271,000	317,000	254,000	337,000	875,000	765,000	982,000	865,000	731,000	1,030,000	221,000	319,000	293,000	300,000	296,140	347,000	344,000	469,000	400,000
MEDP1510	Asp-Phe	**281**	C13H16N2O5	1,950,000	1,790,000	1,800,000	1,620,000	1,990,000	1,910,000	35,100,000	32,900,000	40,000,000	34,300,000	33,800,000	34,400,000	25,900,000	29,200,000	28,600,000	26,900,000	27,967,800	29,200,000	22,100,000	23,600,000	23,200,000
MEDP1511	Met-Glu	**279**	C10H18N2O5S	87,700	83,400	80,000	84,800	78,400	87,100	252,000	220,000	305,000	252,000	173,000	307,000	73,000	77,500	114,000	66,900	85,160	94,100	133,000	130,000	116,000
MEDP1512	Phe-Val	**265**	C14H20N2O3	177,000	169,000	186,000	261,000	184,000	190,000	6,140,000	6,580,000	5,710,000	6,060,000	5,970,000	6,380,000	7,730,000	4,530,000	5,580,000	5,240,000	5,609,500	4,970,000	4,680,000	4,670,000	4,640,000
